# Understanding Macrophage Complexity in Metabolic Dysfunction-Associated Steatotic Liver Disease: Transitioning from the M1/M2 Paradigm to Spatial Dynamics

**DOI:** 10.3390/livers4030033

**Published:** 2024-09-13

**Authors:** Forkan Ahamed, Natalie Eppler, Elizabeth Jones, Yuxia Zhang

**Affiliations:** Department of Pharmacology, Toxicology and Therapeutics, University of Kansas Medical Center, MS 1018, 3901 Rainbow Boulevard, Kansas City, KS 66160, USA

**Keywords:** macrophages, diversity, Kupffer cell, monocyte-derived macrophage, spatial dynamics, metabolic dysfunction-associated steatotic liver disease (MASLD)

## Abstract

Metabolic dysfunction-associated steatotic liver disease (MASLD) encompasses metabolic dysfunction-associated fatty liver (MASL) and metabolic dysfunction-associated steatohepatitis (MASH), with MASH posing a risk of progression to cirrhosis and hepatocellular carcinoma (HCC). The global prevalence of MASLD is estimated at approximately a quarter of the population, with significant healthcare costs and implications for liver transplantation. The pathogenesis of MASLD involves intrahepatic liver cells, extrahepatic components, and immunological aspects, particularly the involvement of macrophages. Hepatic macrophages are a crucial cellular component of the liver and play important roles in liver function, contributing significantly to tissue homeostasis and swift responses during pathophysiological conditions. Recent advancements in technology have revealed the remarkable heterogeneity and plasticity of hepatic macrophage populations and their activation states in MASLD, challenging traditional classification methods like the M1/M2 paradigm and highlighting the coexistence of harmful and beneficial macrophage phenotypes that are dynamically regulated during MASLD progression. This complexity underscores the importance of considering macrophage heterogeneity in therapeutic targeting strategies, including their distinct ontogeny and functional phenotypes. This review provides an overview of macrophage involvement in MASLD progression, combining traditional paradigms with recent insights from single-cell analysis and spatial dynamics. It also addresses unresolved questions and challenges in this area.

## 1. Introduction

The accumulation of excess fat in the liver exceeding 5% can result in steatotic liver disease (SLD). Among various etiologies of steatosis, individuals with minimal or no alcohol consumption were previously diagnosed with a condition known as nonalcoholic fatty liver disease (NAFLD). NAFLD is now referred to as metabolic dysfunction-associated steatotic liver disease (MASLD), encompassing patients with hepatic steatosis and at least one of five cardiometabolic risk factors [1]. MASLD includes two histological subtypes: metabolic dysfunction-associated fatty liver (MASL), a relatively mild form characterized by fat accumulation or steatosis in the liver, and metabolic dysfunction-associated steatohepatitis (MASH), a progressive form accompanied by steatosis, inflammation, hepatocyte death, and fibrosis. A meta-analysis of PubMed/MEDLINE data spanning from 1989 to 2015, focusing on terms related to the epidemiology and progression of MASLD, revealed that approximately over 25% of the global population is now thought to have MASLD, with estimated global prevalence rates for MASH ranging from 1.5% to 6.45% [2]. A recent study using dynamic Markov modeling to assess the burden of MASLD-related disease predicted that by 2030, the total MASLD population will increase, reaching a prevalence of28.4% [3]. MASLD has the potential to progress to cirrhosis and ultimately to hepatocellular carcinoma (HCC) [4,5]. MASLD is now identified as the primary etiology contributing to the incidence of HCC in the US, and the prevalent HCC cases associated with MASLD are estimated to rise, with an anticipated increase from 10,820 in 2016 to 24,860 cases in 2030 [3]. Age, gender, ethnicity, and metabolic conditions like diabetes and obesity are recognized as major risk factors for MASL and MASH. In addition, genetic and environmental factors add to the complexity of MASLD [6–8]. Additionally, MASLD is rapidly becoming the leading cause of liver transplantation in the United States [9]. The economic burden of MASLD is enormous, with the healthcare costs for MASLD patients significantly exceeding those for patients with similar comorbidities but without MASLD [10,11].

Researchers have extensively studied the pathogenesis and progression of MASLD, recognizing the involvement of both intrahepatic liver parenchymal and nonparenchymal cells and extrahepatic components in disease development [12]. One conceptual framework used to explain the progression from MASL to MASH is the ‘two-hit’ hypothesis. This hypothesis proposes that dysregulated hepatic lipid accumulation constitutes the initial hit, while oxidative, metabolic, and cytokine stresses represent the second hit [13,14]. In addition, a ‘three-hit’ hypothesis suggests that in MASLD, oxidative stress diminishes hepatocyte proliferation, prompting alternative regeneration pathways involving hepatic progenitor cells, with fibrosis progression dependent on the efficiency of hepatocyte regeneration, thus implicating impaired progenitor cell proliferation and cell death as the ‘third hit’ in MASLD progression [15–17]. Recently, there has been significant attention on immunological aspects in MASLD progression, particularly the involvement of macrophages. Hepatic macrophages are key players in innate immunity and a crucial cellular component of the liver, with the ratio of hepatocytes to macrophages ranging from 5:1 to 2.5:1 [18]. Macrophages contribute significantly to liver homeostasis, injury, and repair, exhibiting diverse subpopulations that dynamically change in health and disease [19]. In MASLD, macrophages can drive inflammation, fibrosis progression, and regression, thus influencing the disease’s pathogenesis and progression [5,20,21].

Traditionally, macrophages have been classified based on the M1/M2 paradigm, but recent advancements in high-end technologies have rendered this classification outdated, particularly in the context of deciphering the liver macrophage landscape. There has been a rapid expansion in understanding the clinical and research implications of liver macrophage diversity in MASLD over the past 5 years. Additionally, the spatial localization of individual cells is increasingly recognized as a crucial parameter defining their function, necessitating integration into advanced multidimensional analyses. In this review, we summarize the role of macrophages in various stages of MASLD, encompassing the traditional M1/M2 paradigm alongside recent insights from single-cell and spatially resolved analyses. Furthermore, we offer perspectives on unresolved questions and challenges in this field.

## 2. General Overview of Macrophages

Macrophages, a distinct type of cells with a unique ability to clear up foreign bodies such as bacteria, viruses, debris, and other particles through a process known as phagocytosis, were discovered by a Russian-born zoologist and microbiologist Ilya (Elie) Metchnikoff nearly 140 years ago [22]. Metchnikoff’s groundbreaking discovery earned him the Nobel Prize in Physiology and Medicine in 1908. Macrophages are integral components of the mononuclear phagocytic system (MPS), a classification introduced by Furth et al. to encompass phagocytic mononuclear cells, which consist of immature monocytic cells along with their bone-marrow precursors, peripheral blood monocytes, and tissue-resident macrophages [23]. Lineage studies examining gene expression profiles and recruitment dynamics of various tissue macrophages have uncovered distinct developmental origins of these cells. This includes embryonic-derived and monocyte-derived macrophages, and these lineages remain independent of each other throughout adulthood [23–28]. As the effector cells of the innate immune system, macrophages play a crucial role as the body’s primary line of defense. They not only identify and eliminate pathogens but also engage in communication with a specialized defense mechanism known as the adaptive or acquired immune system [29].

Macrophages are ubiquitous across various tissues in the body, serving crucial functions throughout an organism’s life, from developmental stages to maintaining homeostasis and influencing the pathophysiology of diseases. They are motile cells, and their migrations toward the sites of infection and inflammation are critical for their role as effector cells in innate immunity. This migration is mediated by the expression of a diverse array of surface receptors on macrophages, which facilitate their interaction with foreign ligands. These receptors enable macrophages to sense their environment and perform various functions. Typical examples include phosphatidylserine recognition receptors for apoptotic cell removal, complement receptors for clearing opsonized necrotic cells and altered-self molecules, and pattern recognition receptors (PRRs) such as Toll-like receptors (TLRs), nod-like receptors (NLRs), sensors for intracellular DNA and RNA, c-type lectin, and scavenger receptors. This PRR family allows macrophages to recognize and bind directly to pathogens and their products, initiating processes like inflammasome activation [30,31].

Resident macrophages are crucial components of tissues, contributing to organ development and maintaining homeostasis. They adapt to their environment, displaying specialized functions. Despite being fully differentiated, resident macrophages exhibit high plasticity and undergo phenotypic reprogramming in response to a changing tissue microenvironment [25,32]. In a healthy state, resident macrophages balance the response to foreign particles while minimizing tissue damage. They patrol tissues, phagocytose cellular debris, clean the surroundings, and facilitate tissue repair. Consequently, resident macrophages play essential roles throughout an organism’s lifespan, including promoting ductal branching, angiogenesis, vascular remodeling, osteoclast and bone remodeling, erythropoiesis, brain development, and lung homeostasis during early developmental stages [33–37]. For instance, in tissues such as the mammary glands, pancreas, and kidneys, macrophages are essential for clearing apoptotic epithelial cells, regulating cell proliferation during lumen formation, and secreting growth factors and cytokines that promote tissue remodeling and ductal branching. Their absence can result in ductal branching abnormalities in these organs [38]. In mice with null mutations in the colony-stimulating factor 1 (*Csf1*) gene, which leads to the absence of macrophages, various developmental abnormalities occur, such as atrophic mammary glands, reduced mass of insulin-producing β-cells, impaired pancreatic cell proliferation, and compromised kidney function [39,40]. Additionally, the absence of osteoclasts, which are resident macrophages crucial for bone remodeling, leads to the development of osteopetrosis [41]. In specialized bone marrow areas called erythroblastic islands, macrophages support the production of red blood cells by maintaining cell interactions between erythroblasts and macrophages [42,43] and facilitate erythropoiesis by phagocytizing extruded erythroblast nuclei and supplying iron to erythroid progenitors [44,45]. Microglia, the tissue-resident macrophages of the brain and spinal cord play essential roles in central nervous system (CNS) development, homeostasis, and diseases [46,47]. Alveolar macrophages, found in the lungs, help maintain lung function by clearing inhaled dust [48].

## 3. Hepatic Macrophages: Type, Origin, and Function

**Figure 1.**
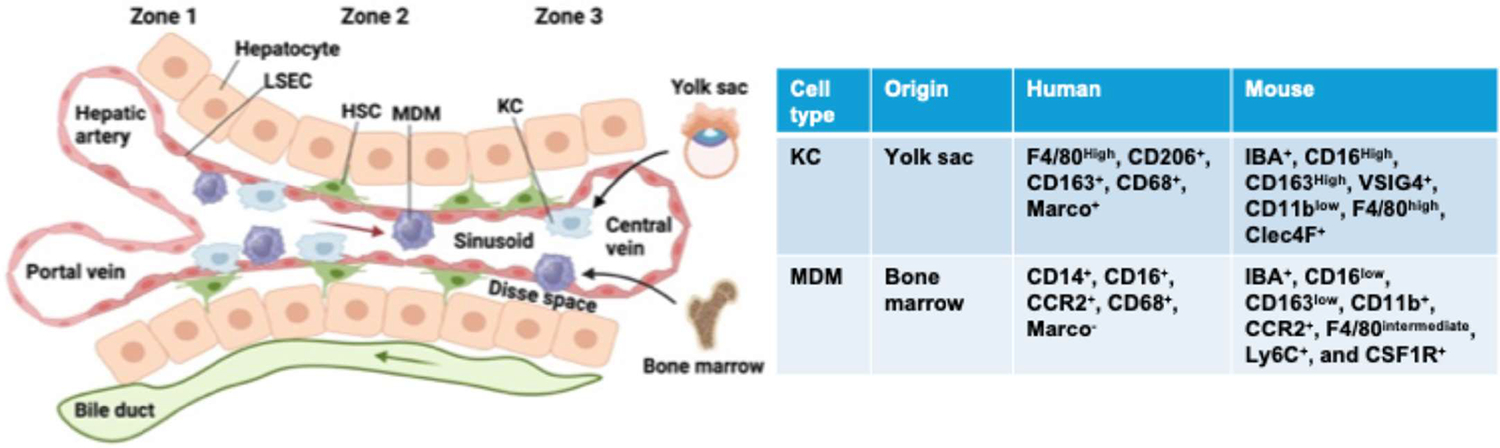
Schematic overview of hepatic sinusoid and macrophages. The liver is divided into three zones: the areas around the hepatic arteries and portal veins are known as zone 1, those near the central vein are zone 3, and the cells in between are referred to as zone 2. Oxygen-rich blood from the hepatic artery combines with nutrient-rich blood from the portal vein and flows along the sinusoids toward the central vein (red arrow). Meanwhile, bile flows from zone 3 to zone 1, collected by the bile ducts (green arrow). Hepatic macrophages consist primarily of two distinct subtypes: liver resident Kupffer cells (KC), originating from yolk sac, and monocyte-derived macrophages (MDM), from the bone marrow (black arrow). KCs and MDMs can be differentiated by their distinct cell surface markers. Located near liver sinusoidal endothelial cells (LSECs) along the hepatic sinusoids, KCs and MDMs play an important role in influencing the activity of hepatic stellate cells (HSCs) and hepatocytes.

Hepatic macrophages consist of two major types: resident macrophages and infiltrated monocyte-derived macrophages (MDM) that rapidly emerge during injury. The resident hepatic macrophages were initially discovered by Karl Wilhelm von Kupffer, a Baltic German anatomist, and are now known as Kupffer cells (KCs) [49]. Comprising approximately 15% of the total liver cell population, KCs represent 80–90% of all tissue-resident macrophages in the body [50]. Within liver lobules, 43% of Kupffer cells are distributed in the periportal area, 28% in the midzonal area, and 29% in the centrilobular area [51]. KCs are located within the lumen of liver sinusoids with proximity to the liver sinusoidal endothelial cells (LSECs) that form the blood vessel walls (Figure 1). Despite being considered fixed resident cells, evidence shows that KCs can exhibit some degree of mobility along sinusoidal walls, either with or against the direction of blood flow [52].

Differential expressions of cell surface markers enable the distinction between KCs and MDMs. In mice, KCs are identified as IBA^+^, CD16^High^, CD163^High^, VSIG4^+^, CD11b^low^, F4/80^high^, and Clec4F^+^, while MDMs are defined as IBA^+^, CD16^low^, CD163^low^, CD11b^+^, CCR2^+^, F4/80^intermediate^, Ly6C^+^, and colony stimulating factor 1 receptor (CSF1R)^+^ [53–56] (Figure 1). A fate mapping experiment revealed that erythro-myeloid progenitors (EMPs), originating in the yolk-sac at embryonic day E8.5 and subsequently colonizing the fetal liver at E10.5, give rise to KCs during embryonic development [57,58]. In one-year-old mice, most KCs are of embryonic origin, with some derived from hematopoietic stem cells [58,59]. The average half-life of mouse KCs is 12.4 days, while in rats, their lifespan extends from several weeks to months [60,61]. KCs are self-renewing and can proliferate into mature cells, making their replenishment in a steady state independent of MDMs [27,62]. Bone marrow progenitor cells, defined as CX3CR1^+^CD117^+^Lin^−^, differentiate into MDMs, which can further be subdivided into Ly6C^low^ or Ly6C^high^ MDMs in mouse models of liver diseases [63].

Studies on mice have provided considerable insights into the tissue residence and replenishment of macrophages in human livers. Remarkably, recent single-cell RNA sequencing of human fetal and adult livers has identified distinct clusters of macrophages, showing some transcriptional overlap with hepatic KCs and infiltrating MDMs defined in mice [64–69]. For instance, a study using single-cell RNA-Seq to analyze transcriptional profiles of parenchymal and non-parenchymal cells from fresh hepatic tissues of five healthy human livers identified two distinct subgroups of intrahepatic CD68^+^ macrophages based on the expression of the macrophage receptor with collagenous structure (MARCO): CD68^+^MARCO^+^ KCs and CD68^+^MARCO^−^ macrophages [64]. This study found that MARCO is expressed exclusively in non-inflammatory KCs, with CD68^+^MARCO^+^ cells concentrated in periportal areas contributing to immunotolerance. In contrast, CD68^+^MARCO^−^ macrophages exhibit a proinflammatory transcriptional profile like that of recruited macrophages found in mouse livers [64].

Unlike mouse KCs, the half-life of human KCs is not precisely defined. Estimates from animal studies and limited human data suggest that the turnover rate of human KCs may be on the order of several months to years, varying depending on factors such as age, health status, and environmental conditions. A recent study using human leukocyte antigen (HLA)-mismatched liver allografts to distinguish donor liver-resident KCs from recipient infiltrating MDMs showed that although allografts were rapidly infiltrated by recipient monocytes that underwent partial reprogramming to macrophages, a small residual pool of donor cells, including KCs, persisted in the allografts for over a decade [70]. This study revealed remarkably long-lived KCs in human livers. Further research is needed to provide a more precise estimation of the average half-life of human KCs.

KCs serve as the liver’s primary defense against pathogens and antigens from the gastrointestinal tract. To maintain tissue homeostasis, the liver fosters an anti-inflammatory microenvironment, promoting immunological tolerance to prevent unnecessary immune responses against food-derived antigens and bacterial products from the portal vein. Among liver cells, KCs play a central role in scavenging circulating antigens. Their immunological tolerance function involves several mechanisms, including presenting antigens to promote the arrest of CD4 T-cells and the expansion of interleukin-10-producing regulatory T cells, fostering tolerogenic immunity [71]. In addition, KCs express lower levels of Major Histocompatibility Complex (MHC) class II and costimulatory molecules such as B7-1 and B7-2, leading to weaker T cell activation compared to dendritic cells [72]. Moreover, KCs generate prostaglandins like PGE2 and 15d-PGJ2, which can suppress the dendritic cell-mediated activation of antigen-specific T cells [72].

## 4. Macrophage Accumulation in MASLD: Insights from Animal Models and Human Studies

In diet-induced mouse models of MASLD, hepatic macrophage numbers increase significantly with the feeding period [73]. For instance, the infiltrated proinflammatory CD45^+^/CD11b^+^/F4/80^intermediate^ MDMs population in the liver of obese mice nearly doubled compared to a lean control [74]. Importantly, diet not only increases the infiltration of MDMs but also disrupts the balance of pro and anti-inflammatory macrophages in the liver [75]. Therefore, characterizing the heterogeneity of hepatic macrophage subpopulations is crucial for advancing our understanding of MASLD.

Resident KCs are the predominant hepatic macrophages in the healthy liver, but their numbers have been reported to be reduced in MASH and MASH-associated HCC [76,77]. As the KC number depletes, MDMs infiltrate the liver [78]. Chemokines are small heparin-binding proteins regulating cell trafficking and play a crucial role in this process. Monocyte chemoattractant protein-1 (MCP-1/CCL2), a member of the C-C chemokine family and a potent chemoattractant for monocytes, controls the migration and infiltration of MDMs. While various cell types produce CCL2, monocytes/macrophages are the major source [79,80]. CCL2 exerts its effects through its cognate receptor CCR2, whose expression is restricted to certain cell types, including monocytes [81]. Studies in both ob/ob and HFD-fed obese mice models have shown a positive association between hepatic expression of Ccl2 and Ccr2 and body weight. In a study by Morinaga et al. [82], both KCs and MDMs were fluorescently labeled and evaluated in a high-fat diet-fed MASH mice model. They found that the MDM population in obese mice was approximately six times higher in number and more proinflammatory compared to MDMs from lean mice. This study demonstrated that KCs played a vital role in recruiting MDMs, which is evident from the significantly elevated expression of CCL2, while MDMs displayed significantly higher expression of CCR2 [82]. This group also demonstrated that blocking the infiltration of MDMs using CCR2 antagonists ameliorated steatohepatitis and fibrosis [83].

Similarly, increased macrophage numbers have been reported in liver samples from MASH patients. A retrospective study analyzing liver biopsies from young MASLD patients has revealed elevated numbers of CD68^+^ KCs, with higher levels correlating with MASLD severity [84]. Another study discovered the presence of enlarged KCs with significantly elevated phagocytic activity in the hepatic sinusoids [85]. These enlarged KCs are closely associated with transformed hepatic stellate cells and oval cells during MASH development. In addition, a significantly increased number of CCR2^+^ MDMs in human liver samples is strongly correlated with the severity of MASH and fibrosis [82]. Furthermore, an increased number of portal macrophages with elevated expression of proinflammatory cytokines IL1B and tumor necrosis factor (TNF) has been observed in patients with MASL progression to MASH [86]. Similar to findings in mouse studies, an increased presence of CD11c-positive macrophages surrounding hepatocytes with large lipid droplets, forming aggregates known as hepatic crown-like structures, correlates with hepatocyte death and fibrosis development in human MASH patients [87,88]. These aggregates are important sources of inflammation and fibrosis due to their intact structure and close association with activated fibroblasts for collagen deposition.

## 5. Stimuli Trigger Hepatic Macrophage Activation during MASLD Development

Multiple stimuli, such as fatty acids, cholesterol, damage-associated molecular patterns (DAMPs), and pathogen-associated molecular patterns (PAMPs), trigger macrophage activation in MASLD. The heightened influx of fatty acids into the liver, along with de novo lipogenesis, exacerbates oxidative stress and lipid peroxidation in MASLD [89]. When co-cultured with macrophages, steatotic hepatocytes release pro-inflammatory cytokines such as TNFα, MCP-1, interleukin 6 (IL-6), and IL-18, which can activate the macrophages [90]. Saturated fatty acids, such as lauric acid (C12:0) and palmitic acid (C16:0), induce the expression of inflammatory markers, including cyclooxygenase-2 (COX-2), inducible nitric oxide synthase (iNOS), and IL-1α in a mouse macrophage cell line Raw 264.7 cells through the activation of TLR4 and NF-κB pathway [91]. On the contrary, unsaturated fatty acids inhibit the NF-κB pathway, thus impeding saturated fatty acid-mediated COX-2 expression in Raw 264.7 cells. In addition, palmitic acid activates TLR2 in a human monocytic cell line THP-1 cells, inducing inflammasome-mediated-IL-1β production [92]. Saturated fatty acids activate macrophages and promote inflammation, while unsaturated fatty acids inhibit it. This has been demonstrated not only in vitro but also in in vivo mouse studies and patients with MASLD. Using dietary mouse models, Kim et al. found that palmitate stimulates reactive oxygen species (ROS) production in CD11b^+^F4/80^low^ infiltrating macrophages rather than resident macrophages [93]. This occurs through the direct binding of palmitate to a monomeric TLR4-MD2 complex, triggering endocytosis of TLR4 and NADPH oxidase 2 (NOX2), leading to pro-interleukin-1β expression in macrophages [93]. Consequently, mice lacking *Nox2* are resistant to high-fat diet-induced MASLD development [93,94]. Consistent with this observation, increased serum levels of soluble NOX2-derived peptides and NOX2-generated oxidative stress have been found to be associated with the severity of liver steatosis in MASLD patients [95].

The disruption of hepatic cholesterol homeostasis and accumulation of free cholesterol in hepatocytes are linked to the pathogenesis of MASH [96]. Ioannou et al. described the presence of free cholesterol in the hepatocytes of MASH patients and diet-induced MASH mice model [97]. They proposed that the aggregation and activation of KCs in ‘crown-like structures’ containing cholesterol crystals around lipid droplets, similar to those previously described in inflamed visceral adipose tissue, are significant indicators of the progression of disease from simple steatosis to MASH [97]. To test this hypothesis, they fed c57bl/6J mice a 15% high-fat diet for 6 months, supplemented with various amounts of dietary cholesterol ranging from 0% to 1%. They revealed that increasing cholesterol led to cholesterol loading in the liver but not in adipose tissues, inducing MASH at a threshold dietary cholesterol concentration of 0.5%, whereas mice on lower-cholesterol diets developed only MASL [98]. Additionally, KCs surrounded dead hepatocytes and processed cholesterol crystal-containing lipid droplets, possibly via lysosomal exocytosis, forming the ‘crown-like structures’. These macrophages stained positively for NLRP3 inflammasome and activated caspase 1 [98], likely due to the phagocytosis of cholesterol crystals by macrophages leading to lysosomal swelling and the release of cathepsin B, a lysosomal protease, which activates the NLRP3 inflammasome, a mechanism similar to that responsible for atherosclerosis pathogenesis [99,100].

In addition, oxidative damage to cellular proteins, lipids, and DNA in hepatocytes generates oxidation-specific epitopes, acting as DAMPs, which interact with macrophage-expressed PRRs such as CD36 and TLR4, thereby initiating various immune responses [101]. For instance, in vitro studies demonstrated that mouse KCs can engulf apoptotic bodies from UV-treated mouse hepatocytes, triggering the production of Fas ligand and tumor necrosis factor α (TNFα) [102]. Extracellular vesicles (EVs) are membrane vesicles derived from various types of cells containing biologically active molecules such as RNAs, proteins, and lipids. EVs released from primary mouse hepatocytes, particularly in response to palmitic acid treatment, contain factors like TNF-related apoptosis-inducing ligands [103]. These EVs induce the expression of IL-1β and IL-6 in mouse bone marrow-derived macrophages. In contrast, EVs from primary rat LSECs suppressed the expression of inflammatory genes in LPS-treated KCs [104]. However, this anti-inflammatory effect was diminished when LSECs were exposed to free fatty acids (FFAs), indicating that EVs from LSECs are important in regulating macrophage activation.

Furthermore, PAMPs such as lipopolysaccharide (LPS) and microbial nucleotides act as danger signals recognized by PRRs on macrophages, triggering inflammatory responses via intracellular signaling pathways [105]. In MASLD, there is an increased influx of gut-derived microbial products into the liver due to changes in gut microorganisms and increased intestinal permeability. This leads to elevated TLRs-mediated immune signaling, contributing to liver inflammation and fibrogenesis [106]. In addition, triglycerides enhance the LPS-mediated expression of proinflammatory mediators such as inducible iNOS, TNFα, IL-1β, and IL-6 in rat KCs, compared to LPS stimulation alone [107]. The inhibition of the NF-κB pathway significantly reduces the potentiating effect of triglycerides on iNOS expression by KCs [107]. Electron microscopic analysis of KCs from high-fat diet-fed mice reveals intracellular lipid droplet accumulation [108]. These fat-laden KCs generate significantly high levels of proinflammatory cytokines and chemokines in response to LPS compared to KCs from chow diet-fed mice.

## 6. Classical M1/M2 Macrophage Paradigm in MASLD Development

**Figure 2.**
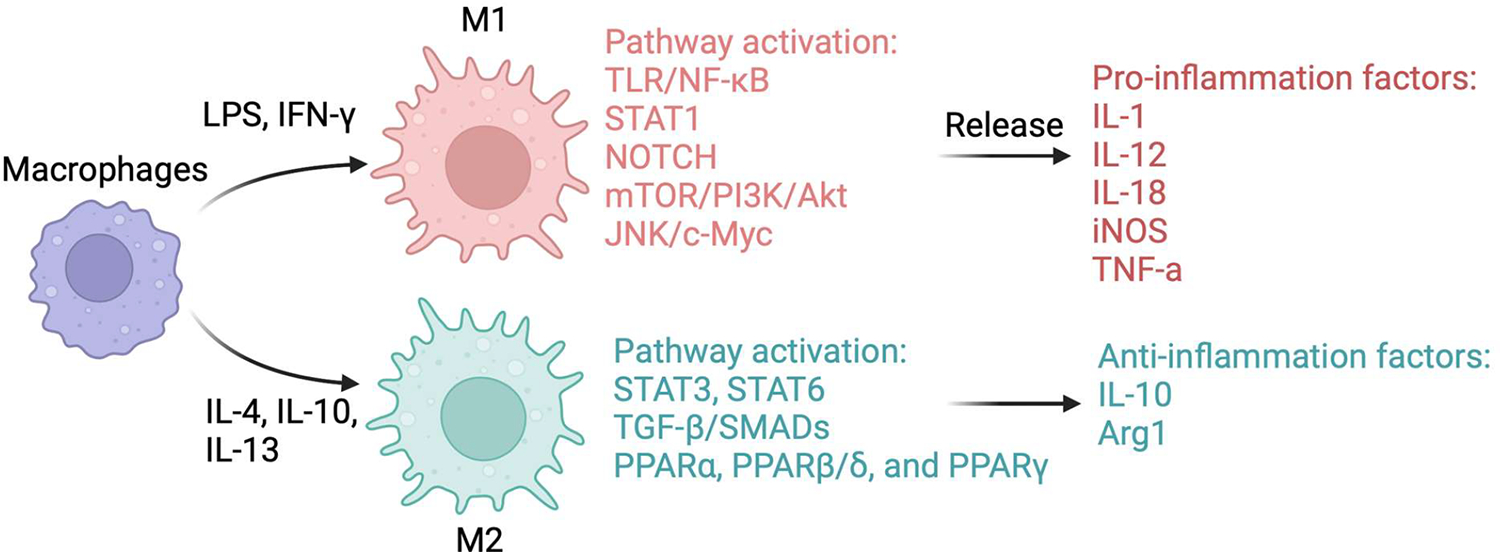
Schematic overview of macrophage M1 and M2 polarization. Macrophages can polarize into two distinct phenotypes depending on the microenvironmental stimuli they encounter. M1 macrophages, induced by LPS and IFN-γ, activate pathways such as TLR/NF-κB, STAT1, NOTCH, mTOR/PI3K/Akt, and JNK/c-Myc, leading to the release of pro-inflammatory factors like IL-1, IL-12, IL-18, iNOS, and TNF-α. In contrast, M2 macrophages, induced by IL-4, IL-10, and IL-13, promote anti-inflammatory activity through pathways like STAT3/6, TGF-β/SMADs, and PPAR (α, β/δ, and γ), expressing IL-10 and arginase 1.

The dynamic heterogeneity and reprogramming of macrophages contribute significantly to disease pathogenesis and progression. An important aspect of this macrophage adaptability is evident in the differentiation of macrophages into either classically activated M1, characterized by a pro-inflammatory profile, or alternatively activated M2 macrophages, displaying anti-inflammatory and pro-fibrogenic phenotypes (73,77). This classification is rooted in their origins from the Th1 strains (C57BL/6, B10D2) or Th2 strains (BALB/c, DBA/2), respectively. Macrophages activated in response to IFN-γ differentiate into M1-like macrophages capable of generating nitric oxide (NO) to kill parasites. On the contrary, Th2 cytokines, including IL-4 and IL-10, suppress the activation of M1-like macrophages, and these M2-like macrophages exhibit elevated arginine metabolism [109,110]. Although the M1/M2 classification oversimplifies the intricate in vivo responses of macrophages, it is widely recognized that the differentiation of macrophages into distinct pro-inflammatory or anti-inflammatory phenotypes profoundly influences host defense and the pathogenesis of various liver diseases. The key mechanisms regulating macrophage polarization are discussed below and illustrated in Figure 2.

### 6.1. Mechanisms Control Macrophage Polarization

#### 6.1.1. TLR and NF-κB

Bacterial endotoxin LPS activates TLR4 on macrophages, triggering proinflammatory reactions crucial for eliminating invading bacteria. Beyond LPS, endogenous DAMPs, including high-mobility group box protein 1 (HMGB1) and hyaluronic acid, are released during tissue injury, activating TLR4 to facilitate tissue repair [111–113]. TLR4 activation leads to NF-κB activation through the myeloid differentiation factor 88 (MyD88)-dependent pathways or interferon regulatory factor (IRF) 3, promoting the expression of proinflammatory factors [114]. Studies have shown that the loss-of-function mutations or deletions of *Tlr4* or *MyD88* protect mice against diet-induced inflammation in adipose tissue and liver, accompanied by altered macrophage polarization [115–117]. In contrast, drugs or compounds inhibiting TLR4/NF-κB signaling can repress M1-like proinflammatory macrophage polarization. For example, Jing et al., using mouse macrophage cell line Raw264.7 cells and primary peritoneal macrophages, demonstrated that berberine, a competitive inhibitor of TLR4, disrupts the TLR4/MyD88/NFκB signaling pathway, interfering with LPS-mediated proinflammatory M1-like macrophage polarization [118]. In a separate study, Xiang et al. showed that olean-28,13β-olide 2 (NZ), a newly synthesized derivative of oleanolic acid, inhibited LPS-mediated generation of proinflammatory cytokines in Raw264.7 cells through the suppression of TLR-NF-κB signaling, downregulation of NLRP3 expression, and inhibition of caspase-1 activation [119]. Similarly, Lu et al. demonstrated that quercetin, a natural flavonoid compound, inhibits LPS-mediated M1 macrophage polarization via the NF-κB and IRF5 signaling [120]. These findings were further supported by in vivo studies showing that berberine and quercetin can inhibit inflammation and prevent metabolic disorders such as MASLD and type 2 diabetes by improving insulin resistance, lipid metabolism, and liver enzymes [121–125]. Together, these findings suggest that the TLR4/NF-κB signaling axis plays an important role in M1-like proinflammatory macrophage polarization, and targeting this pathway holds promise for improving MASLD.

#### 6.1.2. Signal Transducer and Activator of Transcription (STAT)

The STAT family, comprising seven structurally similar and highly conserved members, including STAT1, STAT2, STAT3, STAT4, STAT5A, STAT5B, and STAT6, are recognized as important regulators of macrophage polarization [126,127]. Interferons and TLR signaling polarize macrophages toward the M1-like proinflammatory phenotype via STAT1 signaling, whereas IL-4 and IL-13 tilt macrophages toward the M2-like anti-inflammatory phenotype through STAT6 signaling pathways [128]. In a study with J774 murine macrophages, Haydar et al. demonstrated that azithromycin promotes M2-like macrophage polarization by inhibiting STAT1 and NF-κB signaling pathways. Another study by He et al. showed that IL-4 skews macrophage toward the M2 subtype through the JAK1/STAT6 pathway [129]. In addition, STAT3 plays a determinative role in M2 polarization, as the suppression of JAK3/STAT3 by miR-221-3p promotes the shift of macrophage polarization from the M2 to M1 subtype [130].

#### 6.1.3. Transforming Growth Factor Beta (TGF-β) and Suppressor of Mothers against Decapentaplegic (SMAD)

Upon TGF-β binding to the TGF-β receptor complex, the receptor complex activation triggers Smads (Smad2 or 3)-mediated pathways to regulate gene expression [131]. Studies have revealed the important role of the TGF-β/Smads signaling pathway in modulating M2 macrophage polarization. For instance, growth differentiation factor 3 (GDF3), a member of the TGF-β superfamily, can phosphorylate and activate Smad2/Smad3, inhibiting NLRP3 expression in macrophages and directing macrophage polarization toward the M2 phenotype [132]. The flavonoid compound quercetin can regulate macrophage polarization and reduce kidney fibrosis by antagonizing the TGF-β1/smad2/3 pathway [120]. This inhibitory effect was also observed in a mouse model of asthma-induced airway inflammation, where quercetin treatment reduced airway inflammation response and lung fibrosis by downregulating the TGF-β1/Smad pathway [133].

#### 6.1.4. Peroxisome Proliferator-Activated Receptors (PPARs)

PPARs are a subfamily of nuclear receptors consisting of three members: PPARα, PPARβ/δ, and PPARγ. Their transcriptional activity is mediated by PPAR: retinoid X receptor (RXR) heterodimers, which bind to specific DNA sequence elements called PPREs in the regulatory regions of their target genes. PPARs regulate the expression of genes involved in various functions, including lipid and carbohydrate metabolism, cell proliferation and differentiation, sexual dimorphism, and immune response [134]. PPARα, PPARβ/δ, and PPARγ have tissue-specific but partially overlapping expression patterns. PPARα is highly expressed in tissues that perform significant fatty acid catabolism, such as brown adipose tissue, liver, heart, kidney, and intestine. PPARβ/δ functions prominently in the skin, gut, placenta, skeletal and heart muscles, adipose tissue, and brain. PPARγ exists in two isoforms, PPARγ1 and PPARγ2, which differ at their N termini. PPARγ1 has a broad expression pattern, including the gut, brain, vascular cells, and immune cells, while PPARγ2 is predominantly found in adipose tissues.

All three PPAR isotypes exhibit a common anti-inflammatory function, primarily inhibiting inflammation through transcriptional repression of inflammatory genes. This mechanism involves the activation of PPARs by ligands that bind to key regulators of inflammation, such as NF-kB, activator protein 1 (AP-1), nuclear factor of activated T cells (NFAT), and STAT, resulting in stabilization of corepressor complexes at the promoters of inflammatory genes, thereby repressing their transcription and reducing inflammation [134]. In addition, PPARα has been shown to upregulate the expression of IκB, which prevents the nuclear translocation and activation of NF-κB [135]. Moreover, PPARγ has been shown to directly interact with NF-κB p65, resulting in NF-κB p65 degradation [136].

Studies have underscored the important association between PPAR activation and macrophage polarization [137–139]. For example, PPARα promotes M2 macrophage polarization by interacting with dual specificity phosphatase 1 (DUSP1), which can alleviate cardiomyocyte injury in a macrophage–cardiomyocyte co-culture system [140]. Additionally, delivery of PPARα via lentiviral particles attenuates sepsis-induced myocardial injury in a cecal ligation and puncture mouse model [140]. Activation of PPAR-γ in Raw264.7 macrophages by a PPARγ agonist shifts lipid-mediated macrophage polarization from the M1 to M2 phenotype through its interaction with NF-κB p65 [141]. Similarly, PPARγ activation promotes native human monocytes toward an anti-inflammatory M2 phenotype [142]. Furthermore, a mouse with macrophage-specific deletion of PPARγ impairs the maturation of the M2 macrophage [143]. Eosinophil-derived IL-4 and IL-13 are crucial for maintaining adipose M2 macrophages, which requires PPARβ/δ and PPARγ [144]. These in vitro and in vivo results suggest that PPARs are master regulators of M2 macrophage polarization.

#### 6.1.5. MicroRNAs (miRNAs) and Other Mechanisms

MicroRNAs (miRNAs) have garnered significant interest due to their important roles in macrophage polarization by regulating various signaling pathways [145]. For example, miR-221-3p and miR-1246 facilitate alternative macrophage polarization through modulating JAK3/STAT3 and NF-κB signaling pathways [130,146]. Exosomal vesicles derived from adipocytes delivered miR-34a into macrophages, repressing kruppel-like factor 4 (Klf4) expression and consequently inhibiting M2-like macrophage polarization [147].

Beyond these mechanisms, additional signaling pathways, including Notch signaling, mammalian target of rapamycin (mTOR) signaling, phosphoinositide 3-kinase (PI3K)/Ak strain transforming (Akt), and Jun NH2-terminal kinase (JNK)/c-Myc signaling pathways, have been identified to play roles in macrophage polarization [148–151]. Singla et al. have shown that during M1 macrophage differentiation, the Notch 1 receptor is upregulated, and the activation of Notch signaling promotes THP-1 human monocytes toward M1 macrophage polarization [148]. Conversely, interference with Notch signaling impairs M1 and enhances M2 macrophage polarization [148]. The important role of Notch signaling in macrophage polarization was further supported by an in vivo study showing that myeloid *Notch1* deficiency facilitates M2 macrophage polarization by repressing YAP signaling in an acute liver injury mouse model induced by lipopolysaccharide/D-galactosamine [152]. This study suggests that targeting the macrophage Notch1-YAP circuit could be an effective strategy for treating liver inflammation-related diseases.

The mTOR pathway is a key nutrient/energy sensor that controls cellular metabolism to maintain cellular homeostasis. Dysregulation of mTOR signaling has been implicated in interfering with macrophage polarization and function [153]. This is supported by numerous in vitro and in vivo studies. Mice with myeloid-specific deletion of *Tsc1*, leading to constitutive activation of mTOR complex 1 (mTORC1), exhibit increased susceptibility to sepsis, spontaneous development of inflammatory disorders, and reduced IL-4-induced M2 macrophage polarization, which is accompanied by increased activities of JNK and Ras along with reduced activities of Akt and C/EBPβ [149,154,155]. In line with these studies, myeloid-specific deletion of *Raptor*, which leads to mTORC1 deficiency, protects mice against obesity-induced inflammation and insulin resistance and decreases atherosclerosis development [156,157].

### 6.2. Macrophage Polarization in Early Stage of MASLD

Macrophages with pro-inflammatory phenotypes exacerbate early MASLD severity, while those with anti-inflammatory characteristics contribute beneficially to MASLD initiation. Maina et al., using a methionine-choline-deficient (MCD) diet in C57BL/6 and Balb/c mice, demonstrated that C57BL/6 mice with M1 bias displayed elevated liver steatosis and lobular inflammation compared to Balb/c mice with M2 bias [158]. Further research corroborated that high-fat diet-fed BALB/c mice displayed increased KCs M2 polarization compared to C57BL6/J mice, leading to the apoptosis of M1 KCs via IL10-mediated arginase activation and mitigating liver steatosis and hepatocyte death [159]. Additional studies found that a high-fat diet enriched in polyunsaturated fatty acids promotes alternative M2 macrophage activation and improves metabolic disturbances [159,160]. The activation of M2 KCs by PPARδ promotes and ameliorates obesity-induced insulin resistance [161]. Histidine-rich glycoprotein (HRGP) is an α2-plasma glycoprotein and is mainly produced by liver parenchymal cells in mammals. Liver-derived HRGP has been shown to promote the polarization of M1 macrophages and inhibit M2 polarization in both tumor and inflammatory environments [162]. Consequently, macrophage polarization was tipped toward M2 in mice lacking HRGP, attenuating liver injury and fibrosis induced by MCD diet or carbon tetrachloride (CCl_4_) [163].

### 6.3. Macrophage Polarization in Advanced Stage of MASLD

Liver biopsies from patients with MASH reveal an increase in proinflammatory myeloperoxidase-positive KCs along with elevated expression of the proinflammatory marker IL-6 [164]. Interestingly, the expression of anti-inflammatory macrophage markers such as IL-10 and dectin-1 is also induced in MASH, suggesting a reparative role of M2 macrophages following tissue injury, which may contribute to fibrosis development [87,164]. Type 2 immunity is characterized by increased levels of cytokines such as IL-4, IL-5, IL-9, and IL-13. Blocking anti-inflammatory type 2 TGFβ and IL-13 signaling has been shown to protect against high-fat diet-induced liver fibrosis in mice [165,166]. The scavenger receptor CD163 is considered a marker for anti-inflammatory macrophages. Interestingly, targeting CD163 in KCs and other M2 macrophages with an anti-CD163-IgG-dexamethasone conjugate has been shown to improve MASH pathologies, including hepatic inflammation, hepatocyte ballooning, fibrosis, and glycogen deposition in a rat model of fructose-induced MASH [167]. These findings underscore the important role of M2 macrophage activation in MASLD progression.

## 7. Revealing the Dynamic Landscape of Hepatic Macrophages in MASLD: Heterogeneity and Plasticity

The widespread use of single-cell RNA sequencing (scRNA-seq) has greatly enhanced our comprehension of cellular diversity and changes in macrophage subpopulations under specific healthy or diseased conditions, surpassing the traditional M1/M2 macrophage paradigm. In the normal mouse liver, KCs are identified using markers such as F4/80, CLEC4F, and T cell immunoglobulin and mucin domain-containing 4 (Timd4) [56]. During the early stages of MASLD, KCs engage in lipid storage, compromising their ability for self-renewal [168]. Consequently, embryonic KCs are gradually lost and replaced by MDMs lacking Timd4 expression [169,170]. Mulder K et al. integrated 41 mononuclear phagocyte scRNA-seq datasets to compile a comprehensive monocyte–macrophage-focused compendium, revealing a diverse array of specialized cell subsets distributed across multiple tissues [171]. They identified three conserved macrophage populations across tissues, namely, TREM2, IL4I1, and HES1, suggesting that TREM2 and IL4I1 macrophages could be predominantly derived from monocytes, whereas HES1 macrophages bear an embryonic signature [171]. TREM2 macrophages were initially studied in the context of brain disorders and neurodegeneration; however, recent evidence has revealed their presence not only in the brain but also in adipose tissue, liver, and different types of tumors, indicating a potential immunoregulation role in these contexts [172–174]. Several studies in mice have elucidated the various roles of TREM2^+^ macrophages in liver disease, demonstrating their protective functions for hepatocytes in MASLD and cholangiopathies [175,176], immunosuppressive roles in hepatocellular carcinoma, and contribution to supporting liver regeneration in both acute and chronic murine injury models [173,177]. In another study using scRNA-seq, researchers delineated the functional phenotypes of myeloid cells and liver macrophages throughout the progression of MASH, revealing significant alterations in both liver MDMs and their bone marrow precursors, as indicated by the downregulation of the inflammatory marker calprotectin [178].

Similarly, the transcriptional profiles obtained from an scRNA-seq analysis of parenchymal and non-parenchymal cells in human livers unveil distinct subsets of hepatic macrophages [64]. The first subset, CD68^+^MARCO^−^ macrophages, exhibits characteristics of pro-inflammatory macrophages with an enriched expression of LYZ, CSTA, and CD74. The second subset, CD68^+^MARCO^+^ macrophages, are identified as KCs and expressed genes associated with immune tolerance, including CD5L, MARCO, VSIG4, CD163, MAF, VCAM1, and KLF4. Furthermore, two distinct populations of MARCO^+^ KCs are distinguished by the expression of TIMD4, with a selective reduction in MARCO^+^ TIMD4^−^ KCs observed in the livers of cirrhosis patients [68].

These studies collectively suggest that macrophages exhibit a wider spectrum of phenotypic activation profiles during MASLD development than previously recognized. The integration of single-cell transcriptomics with advanced bioinformatics enables the prediction of novel cellular interactions and macrophage plasticity throughout MASLD progression.

## 8. Unraveling the Complexity of Hepatic Macrophages in MASLD: Insights into Spatial Dynamics

The localization of liver macrophages within hepatic lobules is closely linked to their function. KCs are not restricted to blood vessels but extend into the perisinusoidal space of Disse, where they interact closely with hepatocytes and hepatic stellate cells (HSCs) [170]. Unlike KCs, MDMs are characterized by their smaller size and circulate through the sinusoids [170]. The MDMs’ specific localization around the periportal area suggests that these cells may serve as primary responders to events such as bile duct leakage or the presence of pathogens in the portal vein. Over the past decade, technological advancements such as single-cell analysis and in situ expression measurements of landmark genes have significantly deepened our comprehension of liver macrophage populations during homeostasis and disease. These technologies have revealed spatially specific responses that influence liver disease progression [179–181]. For instance, a 2022 study presented a comprehensive spatial proteogenomic single-cell atlas of the immune cell landscape in healthy and obese human and mouse livers, identifying three distinct macrophage populations in the homeostatic liver, with KCs being the most prevalent [182].

Hepatocyte zonation for metabolic functions is well-known, and recent studies indicate similar spatial variability in macrophages along the centrilobular–portal axis. For instance, during weaning in mice, KCs tend to cluster around periportal regions, influenced by the activation of liver sinusoidal endothelial cells triggered by MYD88-dependent signaling from gut-derived bacteria, suggesting the significance of KC zonation in controlling pathogen dissemination [183]. In addition, spatial transcriptomics of healthy human liver tissues unveils non-inflammatory macrophage genes and signatures in periportal regions and inflammatory counterparts closer to the central vein [184]. During liver injury, the hepatic macrophage landscape undergoes significant changes associated with disease stages. In a study using a mouse model with conditional depletion of liver KCs, researchers demonstrate that Ly6C-high monocytes, when recruited, could differentiate into F4/80^+^ KCs to replenish the KC pool [170]. Their subsequent CSF1R-dependent proliferation reaches the steady-state KC density by day 6 after depletion [170].

Consistent changes in the spatial dynamics of immune cell subsets have been observed during the progression of human MASLD. A summary of studies utilizing scRNAseq, snRNAseq, and spatial transcriptomics on normal human livers and livers from MASLD patients is provided in Table 1 [64–66,68,182,184–194]. For instance, researchers combining single-cell and -nucleus sequencing with spatial mapping have revealed distinct and evolutionarily conserved, spatially restricted hepatic macrophage niches, such as Gpnmb^+^ Spp1^+^ lipid-associated macrophages (LAMs) in the centrilobular areas where steatosis occurs [182]. This study also found that KC development crucially depends on their cross-talk with HSCs via an activin receptor-like kinase (ALK1)–bone morphogenic protein (BMP) 9/10 axis [182]. Another study using human liver samples from patients with MASLD and primary sclerosing cholangitis revealed intense aggregation of IBA1^+^ CD16^low^ CD163^low^ MDM-derived macrophage, exhibiting distinct spatial proximity to CK19^+^ ductular cells in periportal areas [195]. Additionally, the accumulation of IBA1^+^ CD163^low^ MDMs tightly correlates with the loss of hepatocytes and increased ductular reaction during the progression of MASLD, primary sclerosing cholangitis, primary biliary cholangitis, and alcoholic hepatitis [195,196].

Fibrotic liver disease causes significant changes in vascular architecture. KCs, located within liver sinusoids, primarily filter bacteria-rich portal blood flow. To understand how fibrotic remodeling of the vascular architecture affects the KC compartment, a study using high-resolution intravital microscopy (IVM) in a mouse model of liver fibrosis induced by CCl_4_ revealed that increased collagen deposition and collateral vessel growth around sinusoids cause sinusoid-resident KCs to lose their identity and function due to diminished contact with parenchymal cells [197]. This study found that MDMs are recruited and formed multinucleated KC-like syncytia within these collateral vessels. These KC-like syncytia displayed enhanced bacterial capture ability and are also observed in human liver cirrhosis from different etiologies, including cholestatic liver disease, viral hepatitis, alcoholic hepatitis, and MASLD [197]. Taken together, these studies revealed significant changes in the spatial dynamics of macrophage subsets in MASLD and other chronic liver diseases. Future studies will focus on fully understanding how macrophage heterogeneity evolves throughout liver disease progression.

## 9. Targeting Macrophages for the Treatment of MASLD

Macrophages have emerged as important therapeutic targets in MASLD. The impact of Kupffer cells on steatosis, to some extent, is regulated by IL-1beta-mediated suppression of PPARα expression and activity, a master regulator of fatty acid oxidation in the liver [198]. A 2010 study showed that the depletion of resident KCs in rats using gadolinium chloride had a protective role against diet-induced alterations in hepatic lipid metabolism and insulin sensitivity [199].

In monocytes/macrophages and KCs, the glucocorticoid receptor (GR) and glucocorticoid-induced leucine zipper (GILZ) axis is involved in a variety of inflammatory processes, contributing to the pathogenesis of liver inflammation. The knockdown of Gilz renders KCs more susceptible to LPS, and transgenic mice overexpressing macrophage-specific Gilz significantly reduce obesity-induced liver inflammation [200]. Dexamethasone, a potent glucocorticoid widely used to treat diseases including multiple sclerosis, allergies, cerebral edema, inflammation, and shock, has been explored in the context of MASLD treatment. Svendsen et al. demonstrated that a low dose of an anti-CD163-IgG-dexamethasone conjugate, specifically targeting CD163 receptors on KCs and alternatively activated macrophages, significantly reduces high-fructose diet-induced MASH-like pathologies, including hepatocyte ballooning, hepatic inflammation, and fibrosis in rats, without apparent systemic side effects [167].

Therapies targeting or inhibiting pro-fibrotic macrophages have been evaluated in various clinical trials. Drugs like CCR2/CCR5 inhibitors (e.g., cenicriviroc) and galectin-3 antagonists (e.g., GR-MD-02) have shown potential in reducing fibrosis in MASLD [201,202]. For instance, in the CENTAUR trial, year 1 data for cenicriviroc, a CCR2/CCR5 antagonist, revealed fibrosis improvement in 20% of patients without affecting steatohepatitis compared to a placebo, though continued fibrosis improvement was not observed by the end of year 2 [201,203]. Galectin-3 is a member of the endogenous lectin family with the ability to bind to terminal galactose residues in glycoproteins. It modulates immune cell adhesion and migration, cytokine production, phagocytosis, and immune cell survival [204–207]. Mice with Gal-3 deficiency are protected from dietary-induced MASH [208,209], which led to the development of galectin-3 inhibitors as a treatment strategy for MASH. Belapectin (GR-MD-02), a soluble and physiologically compatible polysaccharide derived from a natural plant compound consisting of oligosaccharide chains with galactose residues that specifically bind to galectin-3, holds promise for the treatment of MASH with advanced fibrosis. Traber et al. studied GR-MD-02 in a high-fat diet-fed mouse MASH model and found that it reduced Gal-3 expression in liver macrophages and ameliorated MASH progression [210]. A phase 1 study demonstrated that belapectin is safe and well tolerated at single and multiple doses in patients with well-characterized MASH and advanced fibrosis but not cirrhosis [202]. While a phase 2b trial of belapectin in patients with MASH cirrhosis and portal hypertension conducted throughout 36 centers in the USA did not meet its primary endpoint, a sub-analysis excluding patients with esophageal varices showed that belapectin at 2 mg/kg reduced hepatic vein pressure gradient and the development of new varices [211]. This results in an adaptive phase 2B-3 study of belapectin currently being initiated to evaluate its safety and efficacy among MASH cirrhosis patients without esophageal varices [212].

## 10. Conclusions and Challenges

Hepatic macrophages are essential cellular components of the liver, playing critical roles in maintaining tissue homeostasis and facilitating rapid responses to pathophysiological conditions. Despite significant advances in understanding the biology and role of macrophages in MASLD over recent decades, their heterogeneity and complexity in the disease remain inadequately understood. Most studies have relied on cell surface markers, such as clusters of differentiation, to assess immune cell phenotyping and define cell subpopulations, often assuming specific functions based on marker expression. However, there remains a substantial gap between marker expression and the presumed functions, with limited evidence to demonstrate that macrophages expressing these markers actually perform the associated functions or that cells lacking these markers are functionally inactive.

Developing personalized, macrophage-targeted interventions for MASH treatment continues to present significant challenges for several reasons. Many studies of the hepatic immune response have been conducted in vitro using mouse macrophage cell lines like Raw264.7 or human monocyte cell lines like THP-1, which may not accurately reflect the in vivo responses of hepatic macrophages. This is particularly important, considering that KCs interact with their microenvironment to shape the hepatic cellular landscape and modulate liver function. Additionally, small animal models often fail to reliably translate to human clinical trials. For instance, studies have shown significant differences in gene expression between mouse and human livers in fatty liver disease [213,214]. Guillot et al. reported that monocytic macrophages are key drivers of MASH progression, with marked differences in the spatial localization of recruited monocytes between human MASLD and diet-induced obesity-MASH mouse models [195]. They found that in mouse models of MASH, monocyte-derived macrophages accumulate throughout the liver parenchyma, whereas in human MASH, these macrophages predominantly expand in the portal area, a feature shared by various chronic human liver diseases, including MASLD [195]. These findings underscore the limited predictive value of mouse models for human outcomes. As a result, compounds like the galectin-3 inhibitor belapectin, which shows promise in preclinical mouse models, often fail to demonstrate robust efficacy in improving fibrosis in MASH patients [211]. However, while human studies are more clinically relevant, they also face challenges due to the variability in macrophage populations and druggable targets among patients. Recent research reveals considerable variation in macrophage-related gene and protein expression in MASLD patients, highlighting the importance of accounting for individual differences within the hepatic microenvironment to develop effective treatments [215].

Additionally, the complexity of liver macrophage biology adds further challenges to treatment development. Although various proteins and signaling pathways implicated in macrophage activation in MASLD have been identified, a comprehensive understanding of the coordinated regulatory mechanisms is still lacking. A more detailed examination of how macrophage phenotypes evolve over time could provide insights into their specific roles during distinct phases of the disease, from early steatosis to advanced fibrosis. Liver macrophages represent a heterogeneous population of phagocytes with specific adaptations in their phenotypes to the microenvironment. Therefore, fully capturing the functional contribution of a macrophage population to disease progression or regression in MASLD requires consideration of their spatial contextualization [216]. Given the complexity of the hepatic macrophage landscape and the rapid technological advancements in macrophage biology studies, reaching a consensus on macrophage denominations to decipher the overarching functions of specific subpopulations would be advantageous. Further developments are expected to enable the acquisition of multiple omics data from a single sample on a large scale and with a high number of parameters, such as immunostaining combined with in situ messenger RNA sequencing. These advancements hold the potential to revolutionize our understanding of liver macrophage biology in MASLD.

## Figures and Tables

**Table 1.** Studies on scRNAseq, snRNAseq, and spatial transcriptomics conducted on normal human livers and livers from MASLD patients.

Method	Tissues	Database Accession	Publications
scRNAseq	Fetal livers (10.5- and 17.5-week gestation) and healthy liver tissues from partial hepatectomy of patients with primary or secondary liver tumors or benign liver diseases (*n* = 3)	GSE81252 and GSE96981	[185]
scRNAseq	Livers from donors for liver transplantation (*n* = 5)	GSE115469	[64]
scRNAseq	Non-diseased liver tissues from patients who underwent liver resections for colorectal cancer metastasis or cholangiocarcinoma (*n* = 6)	GSE124395	[66]
scRNAseq	Non-parenchymal cells from healthy livers (*n* = 5) and cirrhotic livers (two MASLD, two ALD, one PBC)	GSE136103	[68]
snRNAseq	Tumor-free liver tissue from a partial hepatectomy of a patient with colon cancer and hepatic metastasis (*n* = 1)	EBI BioStudies S-BSST324	[186]
scRNAseq	Liver CD45^+^ immune cells from healthy livers (*n* = 3)	GSE125188	[65]
scRNAseq	Liver CD45^+^ immune cells from steatosis (*n* = 4) and MASH livers (*n* = 3)	GSE159977	[187]
scRNAseq; Spatial transcriptomics	Fetal livers (8- and 17-week gestation)	GSE167096	[188]
scRNAseq	Liver tissues from donors (*n* = 2)	GSE158723	[189]
scRNAseq	Healthy livers from donors (*n* = 6)	EBI BioStudies E-MTAB-10553	[190]
scRNAseq; snRNAseq; Spatial transcriptomics	Healthy livers from donors for liver transplantation (*n* = 4)	GSE185477	[184]
snRNAseq	Healthy livers (*n* = 2), MASLD cirrhotic livers (*n* = 2), HCC (*n* = 2), and adjacent cirrhotic livers (*n* = 2)	GSE174748 and GSE212047	[191]
scRNAseq; snRNAseq; CITE-seq	Healthy (*n* = 14), >10% steatosis with no fibrosis (*n* = 5)	GSE192742	[182]
scRNAseq	Healthy (*n* = 1) and cirrhotic MASH liver (*n* = 1)	GSE190487 and GSM5724573	[192]
snRNAseq	Normal livers (non-tumor tissue from liver metastasis resections) (*n* = 3) and MASH livers (*n* = 9)	GSE212837	[193]
snRNAseq	Healthy livers (*n* = 3) and MASH livers (*n* = 3)	GSE189600	[194]

## Abbreviations

ARG1: Arginase 1
BMDMs: Bone Marrow-Derived Macrophages
CCL2: Chemokine (C-C Motif) Ligand 2
COL1A1: Collagen Type 1 Alpha 1
COL1A2: Collagen Type 1 Alpha 2
DAMP: Damage-Associated Molecular Patterns
HCC: hepatocellular carcinoma
HSC: hepatic stellate cell
IFNg: Interferon Gamma
IL: interleukin
KC: Kupffer cell
LPS: lipopolysaccharide
MAPK: Mitogen-Activated Protein Kinase
MASH: metabolic dysfunction-associated steatohepatitis
MASL: metabolic dysfunction-associated steatotic liver
MASLD: metabolic dysfunction-associated steatotic liver disease
MCD: Methionine Choline Deficient
MDM: Monocyte Derived Macrophage
NAFL: non-alcoholic fatty liver
NAFLD: nonalcoholic fatty liver disease
NASH: nonalcoholic steatohepatitis
NF-κB: Nuclear Factor Kappa B
NLRP3: NLR Family Pyrin Domain Containing 3
NOS2: Nitric Oxide Synthase 2
NPC: non-parenchymal cell
PA: palmitic acid
PPAR: Peroxisome Proliferator-Activated Receptor
ROS: reactive oxygen species
SLD: steatotic liver disease
T2DM: Type II Diabetes Mellitus
TGF-b: Transforming Growth Factor Beta
TLR: Toll-like receptor
TNFa: Tumor Necrosis Factor Alpha

## References

[1] Rinella ME; Sookoian S From NAFLD to MASLD: Updated naming and diagnosis criteria for fatty liver disease. J. Lipid Res 2024, 65, 100485. 38103785 10.1016/j.jlr.2023.100485 PMC10824973

[2] Younossi ZM; Koenig AB; Abdelatif D; Fazel Y; Henry L; Wymer M Global epidemiology of nonalcoholic fatty liver disease-Meta-analytic assessment of prevalence, incidence, and outcomes. Hepatology 2016, 64, 73–84. 26707365 10.1002/hep.28431

[3] Estes C; Anstee QM; Arias-Loste MT; Bantel H; Bellentani S; Caballeria J; Colombo M; Craxi A; Crespo J; Day CP; Modeling NAFLD disease burden in China, France, Germany, Italy, Japan, Spain, United Kingdom, and United States for the period 2016–2030. J. Hepatol 2018, 69, 896–904. 29886156 10.1016/j.jhep.2018.05.036

[4] Younossi Z; Tacke F; Arrese M; Chander Sharma B; Mostafa I; Bugianesi E; Wai-Sun Wong V; Yilmaz Y; George J; Fan J; Global Perspectives on Nonalcoholic Fatty Liver Disease and Nonalcoholic Steatohepatitis. Hepatology 2019, 69, 2672–2682. 30179269 10.1002/hep.30251

[5] Schuppan D; Surabattula R; Wang XY Determinants of fibrosis progression and regression in NASH. J. Hepatol 2018, 68, 238–250. 29154966 10.1016/j.jhep.2017.11.012

[6] Williams CD; Stengel J; Asike MI; Torres DM; Shaw J; Contreras M; Landt CL; Harrison SA Prevalence of nonalcoholic fatty liver disease and nonalcoholic steatohepatitis among a largely middle-aged population utilizing ultrasound and liver biopsy: A prospective study. Gastroenterology 2011, 140, 124–131. 20858492 10.1053/j.gastro.2010.09.038

[7] Sayiner M; Koenig A; Henry L; Younossi ZM Epidemiology of Nonalcoholic Fatty Liver Disease and Nonalcoholic Steatohepatitis in the United States and the Rest of the World. Clin. Liver Dis 2016, 20, 205–214. 27063264 10.1016/j.cld.2015.10.001

[8] Walker RW; Le KA; Davis J; Alderete TL; Cherry R; Lebel S; Goran MI High rates of fructose malabsorption are associated with reduced liver fat in obese African Americans. J. Am. Coll. Nutr 2012, 31, 369–374. 23529994 10.1080/07315724.2012.10720445

[9] Wong RJ; Aguilar M; Cheung R; Perumpail RB; Harrison SA; Younossi ZM; Ahmed A Nonalcoholic steatohepatitis is the second leading etiology of liver disease among adults awaiting liver transplantation in the United States. Gastroenterology 2015, 148, 547–555. 25461851 10.1053/j.gastro.2014.11.039

[10] Younossi ZM; Blissett D; Blissett R; Henry L; Stepanova M; Younossi Y; Racila A; Hunt S; Beckerman R The economic and clinical burden of nonalcoholic fatty liver disease in the United States and Europe. Hepatology 2016, 64, 1577–1586. 27543837 10.1002/hep.28785

[11] Younossi ZM; Tampi R; Priyadarshini M; Nader F; Younossi IM; Racila A Burden of Illness and Economic Model for Patients with Nonalcoholic Steatohepatitis in the United States. Hepatology 2019, 69, 564–572. 30180285 10.1002/hep.30254

[12] Magee N; Zou A; Zhang Y Pathogenesis of Nonalcoholic Steatohepatitis: Interactions between Liver Parenchymal and Nonparenchymal Cells. Biomed. Res. Int 2016, 2016, 5170402. 27822476 10.1155/2016/5170402 PMC5086374

[13] Day CP; James OF Steatohepatitis: A tale of two “hits”? Gastroenterology 1998, 114, 842–845. 9547102 10.1016/s0016-5085(98)70599-2

[14] Day CP From fat to inflammation. Gastroenterology 2006, 130, 207–210. 16401483 10.1053/j.gastro.2005.11.017

[15] Jou J; Choi SS; Diehl AM Mechanisms of disease progression in nonalcoholic fatty liver disease. Semin. Liver Dis 2008, 28, 370–379. 18956293 10.1055/s-0028-1091981

[16] Dowman JK; Tomlinson JW; Newsome PN Pathogenesis of non-alcoholic fatty liver disease. QJM 2010, 103, 71–83. 19914930 10.1093/qjmed/hcp158 PMC2810391

[17] Roskams T; Yang SQ; Koteish A; Durnez A; DeVos R; Huang X; Achten R; Verslype C; Diehl AM Oxidative stress and oval cell accumulation in mice and humans with alcoholic and nonalcoholic fatty liver disease. Am. J. Pathol 2003, 163, 1301–1311. 14507639 10.1016/S0002-9440(10)63489-X PMC1868311

[18] Lopez BG; Tsai MS; Baratta JL; Longmuir KJ; Robertson RT Characterization of Kupffer cells in livers of developing mice. Comp. Hepatol 2011, 10, 2. 21749715 10.1186/1476-5926-10-2 PMC3148529

[19] Tacke F; Zimmermann HW Macrophage heterogeneity in liver injury and fibrosis. J. Hepatol 2014, 60, 1090–1096. 24412603 10.1016/j.jhep.2013.12.025

[20] Arrese M; Cabrera D; Kalergis AM; Feldstein AE Innate Immunity and Inflammation in NAFLD/NASH. Dig. Dis. Sci 2016, 61, 1294–1303. 26841783 10.1007/s10620-016-4049-x PMC4948286

[21] Sutti S; Bruzzi S; Albano E The role of immune mechanisms in alcoholic and nonalcoholic steatohepatitis: A 2015 update. Expert Rev. Gastroenterol. Hepatol 2016, 10, 243–253. 26634783 10.1586/17474124.2016.1111758

[22] Gordon S Elie Metchnikoff: Father of natural immunity. Eur. J. Immunol 2008, 38, 3257–3264. 19039772 10.1002/eji.200838855

[23] van Furth R; Cohn ZA; Hirsch JG; Humphrey JH; Spector WG; Langevoort HL The mononuclear phagocyte system: A new classification of macrophages, monocytes, and their precursor cells. Bull. World Health Organ 1972, 46, 845–852. 4538544 PMC2480884

[24] Hume DA The mononuclear phagocyte system. Curr. Opin. Immunol 2006, 18, 49–53. 16338128 10.1016/j.coi.2005.11.008

[25] Gautier EL; Shay T; Miller J; Greter M; Jakubzick C; Ivanov S; Helft J; Chow A; Elpek KG; Gordonov S; Gene-expression profiles and transcriptional regulatory pathways that underlie the identity and diversity of mouse tissue macrophages. Nat. Immunol 2012, 13, 1118–1128. 23023392 10.1038/ni.2419 PMC3558276

[26] Wynn TA; Chawla A; Pollard JW Macrophage biology in development, homeostasis and disease. Nature 2013, 496, 445–455. 23619691 10.1038/nature12034 PMC3725458

[27] Schulz C; Gomez Perdiguero E; Chorro L; Szabo-Rogers H; Cagnard N; Kierdorf K; Prinz M; Wu B; Jacobsen SE; Pollard JW; A lineage of myeloid cells independent of Myb and hematopoietic stem cells. Science 2012, 336, 86–90. 22442384 10.1126/science.1219179

[28] Yona S; Kim KW; Wolf Y; Mildner A; Varol D; Breker M; Strauss-Ayali D; Viukov S; Guilliams M; Misharin A; Fate mapping reveals origins and dynamics of monocytes and tissue macrophages under homeostasis. Immunity 2013, 38, 79–91. 23273845 10.1016/j.immuni.2012.12.001 PMC3908543

[29] Hirayama D; Iida T; Nakase H The Phagocytic Function of Macrophage-Enforcing Innate Immunity and Tissue Homeostasis. Int. J. Mol. Sci 2017, 19, 92. 29286292 10.3390/ijms19010092 PMC5796042

[30] Ley K; Pramod AB; Croft M; Ravichandran KS; Ting JP How Mouse Macrophages Sense What Is Going On. Front. Immunol 2016, 7, 204. 27313577 10.3389/fimmu.2016.00204 PMC4890338

[31] Gordon S Phagocytosis: The Legacy of Metchnikoff. Cell 2016, 166, 1065–1068. 27565334 10.1016/j.cell.2016.08.017

[32] Biswas SK; Mantovani A Macrophage plasticity and interaction with lymphocyte subsets: Cancer as a paradigm. Nat. Immunol 2010, 11, 889–896. 20856220 10.1038/ni.1937

[33] Park MD; Silvin A; Ginhoux F; Merad M Macrophages in health and disease. Cell 2022, 185, 4259–4279. 36368305 10.1016/j.cell.2022.10.007 PMC9908006

[34] Cohen M; Giladi A; Gorki AD; Solodkin DG; Zada M; Hladik A; Miklosi A; Salame TM; Halpern KB; David E; Lung Single-Cell Signaling Interaction Map Reveals Basophil Role in Macrophage Imprinting. Cell 2018, 175, 1031–1044.e18. 30318149 10.1016/j.cell.2018.09.009

[35] Amit I; Winter DR; Jung S The role of the local environment and epigenetics in shaping macrophage identity and their effect on tissue homeostasis. Nat. Immunol 2016, 17, 18–25. 26681458 10.1038/ni.3325

[36] Gosselin D; Link VM; Romanoski CE; Fonseca GJ; Eichenfield DZ; Spann NJ; Stender JD; Chun HB; Garner H; Geissmann F; Environment drives selection and function of enhancers controlling tissue-specific macrophage identities. Cell 2014, 159, 1327–1340. 25480297 10.1016/j.cell.2014.11.023 PMC4364385

[37] Lavin Y; Merad M Macrophages: Gatekeepers of tissue integrity. Cancer Immunol. Res 2013, 1, 201–209. 24777851 10.1158/2326-6066.CIR-13-0117 PMC4144820

[38] Pollard JW Trophic macrophages in development and disease. Nat. Rev. Immunol 2009, 9, 259–270. 19282852 10.1038/nri2528 PMC3648866

[39] Van Nguyen A; Pollard JW Colony stimulating factor-1 is required to recruit macrophages into the mammary gland to facilitate mammary ductal outgrowth. Dev. Biol 2002, 247, 11–25. 12074549 10.1006/dbio.2002.0669

[40] Banaei-Bouchareb L; Gouon-Evans V; Samara-Boustani D; Castellotti MC; Czernichow P; Pollard JW; Polak M Insulin cell mass is altered in Csf1op/Csf1op macrophage-deficient mice. J. Leukoc. Biol 2004, 76, 359–367. 15178709 10.1189/jlb.1103591

[41] Wiktor-Jedrzejczak W; Bartocci A; Ferrante AW Jr.; Ahmed-Ansari A; Sell KW; Pollard JW; Stanley ER Total absence of colony-stimulating factor 1 in the macrophage-deficient osteopetrotic (op/op) mouse. Proc. Natl. Acad. Sci. USA 1990, 87, 4828–4832. 2191302 10.1073/pnas.87.12.4828 PMC54211

[42] Chasis JA; Mohandas N Erythroblastic islands: Niches for erythropoiesis. Blood 2008, 112, 470–478. 18650462 10.1182/blood-2008-03-077883 PMC2481536

[43] Sadahira Y; Yoshino T; Monobe Y Very late activation antigen 4-vascular cell adhesion molecule 1 interaction is involved in the formation of erythroblastic islands. J. Exp. Med 1995, 181, 411–415. 7528776 10.1084/jem.181.1.411 PMC2191848

[44] Bessis MC; Breton-Gorius J Iron metabolism in the bone marrow as seen by electron microscopy: A critical review. Blood 1962, 19, 635–663. 13868561

[45] Skutelsky E; Danon D On the expulsion of the erythroid nucleus and its phagocytosis. Anat. Rec 1972, 173, 123–126. 5028062 10.1002/ar.1091730111

[46] Colonna M; Butovsky O Microglia Function in the Central Nervous System during Health and Neurodegeneration. Annu. Rev. Immunol 2017, 35, 441–468. 28226226 10.1146/annurev-immunol-051116-052358 PMC8167938

[47] Erblich B; Zhu L; Etgen AM; Dobrenis K; Pollard JW Absence of colony stimulation factor-1 receptor results in loss of microglia, disrupted brain development and olfactory deficits. PLoS ONE 2011, 6, e26317. 22046273 10.1371/journal.pone.0026317 PMC3203114

[48] Whitsett JA; Wert SE; Weaver TE Alveolar surfactant homeostasis and the pathogenesis of pulmonary disease. Annu. Rev. Med 2010, 61, 105–119. 19824815 10.1146/annurev.med.60.041807.123500 PMC4127631

[49] Wake K Karl Wilhelm Kupffer and His Contributions to Modern Hepatology. Comp. Hepatol 2004, 3 (Suppl. S1), S2. 14960154 10.1186/1476-5926-2-S1-S2 PMC2410225

[50] Bilzer M; Roggel F; Gerbes AL Role of Kupffer cells in host defense and liver disease. Liver Int 2006, 26, 1175–1186. 17105582 10.1111/j.1478-3231.2006.01342.x

[51] Bouwens L; Baekeland M; De Zanger R; Wisse E Quantitation, tissue distribution and proliferation kinetics of Kupffer cells in normal rat liver. Hepatology 1986, 6, 718–722. 3733004 10.1002/hep.1840060430

[52] MacPhee PJ; Schmidt EE; Groom AC Evidence for Kupffer cell migration along liver sinusoids, from high-resolution in vivo microscopy. Am. J. Physiol 1992, 263, G17–G23. 1636711 10.1152/ajpgi.1992.263.1.G17

[53] Wen Y; Lambrecht J; Ju C; Tacke F Hepatic macrophages in liver homeostasis and diseases-diversity, plasticity and therapeutic opportunities. Cell. Mol. Immunol 2021, 18, 45–56. 33041338 10.1038/s41423-020-00558-8 PMC7852525

[54] Krenkel O; Tacke F Liver macrophages in tissue homeostasis and disease. Nat. Rev. Immunol 2017, 17, 306–321. 28317925 10.1038/nri.2017.11

[55] Zigmond E; Samia-Grinberg S; Pasmanik-Chor M; Brazowski E; Shibolet O; Halpern Z; Varol C Infiltrating monocyte-derived macrophages and resident kupffer cells display different ontogeny and functions in acute liver injury. J. Immunol 2014, 193, 344–353. 24890723 10.4049/jimmunol.1400574

[56] Scott CL; Zheng F; De Baetselier P; Martens L; Saeys Y; De Prijck S; Lippens S; Abels C; Schoonooghe S; Raes G; Bone marrow-derived monocytes give rise to self-renewing and fully differentiated Kupffer cells. Nat. Commun 2016, 7, 10321. 26813785 10.1038/ncomms10321 PMC4737801

[57] Kim KW; Zhang N; Choi K; Randolph GJ Homegrown Macrophages. Immunity 2016, 45, 468–470. 27653599 10.1016/j.immuni.2016.09.006

[58] Hoeffel G; Chen J; Lavin Y; Low D; Almeida FF; See P; Beaudin AE; Lum J; Low I; Forsberg EC; C-Myb(+) erythro-myeloid progenitor-derived fetal monocytes give rise to adult tissue-resident macrophages. Immunity 2015, 42, 665–678. 25902481 10.1016/j.immuni.2015.03.011 PMC4545768

[59] Gomez Perdiguero E; Klapproth K; Schulz C; Busch K; Azzoni E; Crozet L; Garner H; Trouillet C; de Bruijn MF; Geissmann F; Tissue-resident macrophages originate from yolk-sac-derived erythro-myeloid progenitors. Nature 2015, 518, 547–551. 25470051 10.1038/nature13989 PMC5997177

[60] Bouwens L; Knook DL; Wisse E Local proliferation and extrahepatic recruitment of liver macrophages (Kupffer cells) in partial-body irradiated rats. J. Leukoc. Biol 1986, 39, 687–697. 3458859 10.1002/jlb.39.6.687

[61] Wacker HH; Radzun HJ; Parwaresch MR Kinetics of Kupffer cells as shown by parabiosis and combined autoradiographic/immunohistochemical analysis. Virchows Arch. B Cell Pathol. Incl. Mol. Pathol 1986, 51, 71–78. 2873680 10.1007/BF02899017

[62] Yamamoto T; Naito M; Moriyama H; Umezu H; Matsuo H; Kiwada H; Arakawa M Repopulation of murine Kupffer cells after intravenous administration of liposome-encapsulated dichloromethylene diphosphonate. Am. J. Pathol 1996, 149, 1271–1286. 8863675 PMC1865179

[63] Fogg DK; Sibon C; Miled C; Jung S; Aucouturier P; Littman DR; Cumano A; Geissmann F A clonogenic bone marrow progenitor specific for macrophages and dendritic cells. Science 2006, 311, 83–87. 16322423 10.1126/science.1117729

[64] MacParland SA; Liu JC; Ma XZ; Innes BT; Bartczak AM; Gage BK; Manuel J; Khuu N; Echeverri J; Linares I; Single cell RNA sequencing of human liver reveals distinct intrahepatic macrophage populations. Nat. Commun 2018, 9, 4383. 30348985 10.1038/s41467-018-06318-7 PMC6197289

[65] Zhao J; Zhang S; Liu Y; He X; Qu M; Xu G; Wang H; Huang M; Pan J; Liu Z; Single-cell RNA sequencing reveals the heterogeneity of liver-resident immune cells in human. Cell Discov 2020, 6, 22. 32351704 10.1038/s41421-020-0157-z PMC7186229

[66] Aizarani N; Saviano A; Sagar; Mailly L; Durand S; Herman JS; Pessaux P; Baumert TF; Grun D A human liver cell atlas reveals heterogeneity and epithelial progenitors. Nature 2019, 572, 199–204. 31292543 10.1038/s41586-019-1373-2 PMC6687507

[67] Matchett KP; Wilson-Kanamori JR; Portman JR; Kapourani CA; Fercoq F; May S; Zajdel E; Beltran M; Sutherland EF; Mackey JBG; Multimodal decoding of human liver regeneration. Nature 2024, 630, 158–165. 38693268 10.1038/s41586-024-07376-2 PMC11153152

[68] Ramachandran P; Dobie R; Wilson-Kanamori JR; Dora EF; Henderson BEP; Luu NT; Portman JR; Matchett KP; Brice M; Marwick JA; Resolving the fibrotic niche of human liver cirrhosis at single-cell level. Nature 2019, 575, 512–518. 31597160 10.1038/s41586-019-1631-3 PMC6876711

[69] Popescu DM; Botting RA; Stephenson E; Green K; Webb S; Jardine L; Calderbank EF; Polanski K; Goh I; Efremova M; Decoding human fetal liver haematopoiesis. Nature 2019, 574, 365–371. 31597962 10.1038/s41586-019-1652-y PMC6861135

[70] Pallett LJ; Burton AR; Amin OE; Rodriguez-Tajes S; Patel AA; Zakeri N; Jeffery-Smith A; Swadling L; Schmidt NM; Baiges A; Longevity and replenishment of human liver-resident memory T cells and mononuclear phagocytes. J. Exp. Med 2020, 217, e20200050. 32602903 10.1084/jem.20200050 PMC7478732

[71] Heymann F; Peusquens J; Ludwig-Portugall I; Kohlhepp M; Ergen C; Niemietz P; Martin C; van Rooijen N; Ochando JC; Randolph GJ; Liver inflammation abrogates immunological tolerance induced by Kupffer cells. Hepatology 2015, 62, 279–291. 25810240 10.1002/hep.27793

[72] You Q; Cheng L; Kedl RM; Ju C Mechanism of T cell tolerance induction by murine hepatic Kupffer cells. Hepatology 2008, 48, 978–990. 18712788 10.1002/hep.22395 PMC2600585

[73] Magee N; Ahamed F; Eppler N; Jones E; Ghosh P; He L; Zhang Y Hepatic transcriptome profiling reveals early signatures associated with disease transition from non-alcoholic steatosis to steatohepatitis. Liver Res 2022, 6, 238–250. 36864891 10.1016/j.livres.2022.11.001 PMC9977163

[74] Obstfeld AE; Sugaru E; Thearle M; Francisco AM; Gayet C; Ginsberg HN; Ables EV; Ferrante AW Jr. C-C chemokine receptor 2 (CCR2) regulates the hepatic recruitment of myeloid cells that promote obesity-induced hepatic steatosis. Diabetes 2010, 59, 916–925. 20103702 10.2337/db09-1403 PMC2844839

[75] Ni Y; Nagashimada M; Zhuge F; Zhan L; Nagata N; Tsutsui A; Nakanuma Y; Kaneko S; Ota T Astaxanthin prevents and reverses diet-induced insulin resistance and steatohepatitis in mice: A comparison with vitamin E. Sci. Rep 2015, 5, 17192. 26603489 10.1038/srep17192 PMC4658633

[76] Devisscher L; Scott CL; Lefere S; Raevens S; Bogaerts E; Paridaens A; Verhelst X; Geerts A; Guilliams M; Van Vlierberghe H Non-alcoholic steatohepatitis induces transient changes within the liver macrophage pool. Cell. Immunol 2017, 322, 74–83. 29111158 10.1016/j.cellimm.2017.10.006

[77] Lefere S; Degroote H; Van Vlierberghe H; Devisscher L Unveiling the depletion of Kupffer cells in experimental hepatocarcinogenesis through liver macrophage subtype-specific markers. J. Hepatol 2019, 71, 631–633. 31213365 10.1016/j.jhep.2019.03.016

[78] Tosello-Trampont AC; Landes SG; Nguyen V; Novobrantseva TI; Hahn YS Kuppfer cells trigger nonalcoholic steatohepatitis development in diet-induced mouse model through tumor necrosis factor-alpha production. J. Biol. Chem 2012, 287, 40161–40172. 23066023 10.1074/jbc.M112.417014 PMC3504730

[79] Deshmane SL; Kremlev S; Amini S; Sawaya BE Monocyte chemoattractant protein-1 (MCP-1): An overview. J. Interferon Cytokine Res 2009, 29, 313–326. 19441883 10.1089/jir.2008.0027 PMC2755091

[80] Singh S; Anshita D; Ravichandiran V MCP-1: Function, regulation, and involvement in disease. Int. Immunopharmacol 2021, 101, 107598. 34233864 10.1016/j.intimp.2021.107598 PMC8135227

[81] Wu Y; Ma Y CCL2-CCR2 signaling axis in obesity and metabolic diseases. J. Cell. Physiol 2024, 239, e31192. 38284280 10.1002/jcp.31192

[82] Morinaga H; Mayoral R; Heinrichsdorff J; Osborn O; Franck N; Hah N; Walenta E; Bandyopadhyay G; Pessentheiner AR; Chi TJ; Characterization of distinct subpopulations of hepatic macrophages in HFD/obese mice. Diabetes 2015, 64, 1120–1130. 25315009 10.2337/db14-1238 PMC4375077

[83] Krenkel O; Puengel T; Govaere O; Abdallah AT; Mossanen JC; Kohlhepp M; Liepelt A; Lefebvre E; Luedde T; Hellerbrand C; Therapeutic inhibition of inflammatory monocyte recruitment reduces steatohepatitis and liver fibrosis. Hepatology 2018, 67, 1270–1283. 28940700 10.1002/hep.29544

[84] Park JW; Jeong G; Kim SJ; Kim MK; Park SM Predictors reflecting the pathological severity of non-alcoholic fatty liver disease: Comprehensive study of clinical and immunohistochemical findings in younger Asian patients. J. Gastroenterol. Hepatol 2007, 22, 491–497. 17376039 10.1111/j.1440-1746.2006.04758.x

[85] Lotowska JM; Sobaniec-Lotowska ME; Lebensztejn DM The role of Kupffer cells in the morphogenesis of nonalcoholic steatohepatitis—Ultrastructural findings. The first report in pediatric patients. Scand. J. Gastroenterol 2013, 48, 352–357. 23268566 10.3109/00365521.2012.746390

[86] Gadd VL; Skoien R; Powell EE; Fagan KJ; Winterford C; Horsfall L; Irvine K; Clouston AD The portal inflammatory infiltrate and ductular reaction in human nonalcoholic fatty liver disease. Hepatology 2014, 59, 1393–1405. 24254368 10.1002/hep.26937

[87] Kazankov K; Jorgensen SMD; Thomsen KL; Moller HJ; Vilstrup H; George J; Schuppan D; Gronbaek H The role of macrophages in nonalcoholic fatty liver disease and nonalcoholic steatohepatitis. Nat. Rev. Gastroenterol. Hepatol 2019, 16, 145–159. 30482910 10.1038/s41575-018-0082-x

[88] Itoh M; Kato H; Suganami T; Konuma K; Marumoto Y; Terai S; Sakugawa H; Kanai S; Hamaguchi M; Fukaishi T; Hepatic crown-like structure: A unique histological feature in non-alcoholic steatohepatitis in mice and humans. PLoS ONE 2013, 8, e82163. 24349208 10.1371/journal.pone.0082163 PMC3859576

[89] Neuschwander-Tetri BA Hepatic lipotoxicity and the pathogenesis of nonalcoholic steatohepatitis: The central role of non-triglyceride fatty acid metabolites. Hepatology 2010, 52, 774–788. 20683968 10.1002/hep.23719

[90] Pan X; Wang P; Luo J; Wang Z; Song Y; Ye J; Hou X Adipogenic changes of hepatocytes in a high-fat diet-induced fatty liver mice model and non-alcoholic fatty liver disease patients. Endocrine 2015, 48, 834–847. 25138963 10.1007/s12020-014-0384-x

[91] Lee JY; Sohn KH; Rhee SH; Hwang D Saturated fatty acids, but not unsaturated fatty acids, induce the expression of cyclooxygenase-2 mediated through Toll-like receptor 4. J. Biol. Chem 2001, 276, 16683–16689. 11278967 10.1074/jbc.M011695200

[92] Snodgrass RG; Huang S; Choi IW; Rutledge JC; Hwang DH Inflammasome-mediated secretion of IL-1beta in human monocytes through TLR2 activation; modulation by dietary fatty acids. J. Immunol 2013, 191, 4337–4347. 24043885 10.4049/jimmunol.1300298 PMC3825708

[93] Kim SY; Jeong JM; Kim SJ; Seo W; Kim MH; Choi WM; Yoo W; Lee JH; Shim YR; Yi HS; Pro-inflammatory hepatic macrophages generate ROS through NADPH oxidase 2 via endocytosis of monomeric TLR4-MD2 complex. Nat. Commun 2017, 8, 2247. 29269727 10.1038/s41467-017-02325-2 PMC5740170

[94] Mukherjee R; Moreno-Fernandez ME; Giles DA; Cappelletti M; Stankiewicz TE; Chan CC; Divanovic S Nicotinamide adenine dinucleotide phosphate (reduced) oxidase 2 modulates inflammatory vigor during nonalcoholic fatty liver disease progression in mice. Hepatol. Commun 2018, 2, 546–560. 29761170 10.1002/hep4.1162 PMC5944572

[95] Del Ben M; Polimeni L; Carnevale R; Bartimoccia S; Nocella C; Baratta F; Loffredo L; Pignatelli P; Violi F; Angelico F NOX2-generated oxidative stress is associated with severity of ultrasound liver steatosis in patients with non-alcoholic fatty liver disease. BMC Gastroenterol. 2014, 14, 81. 24758604 10.1186/1471-230X-14-81 PMC4014405

[96] Musso G; Gambino R; Cassader M Cholesterol metabolism and the pathogenesis of non-alcoholic steatohepatitis. Prog. Lipid Res 2013, 52, 175–191. 23206728 10.1016/j.plipres.2012.11.002

[97] Ioannou GN; Haigh WG; Thorning D; Savard C Hepatic cholesterol crystals and crown-like structures distinguish NASH from simple steatosis. J. Lipid Res 2013, 54, 1326–1334. 23417738 10.1194/jlr.M034876 PMC3622327

[98] Ioannou GN; Subramanian S; Chait A; Haigh WG; Yeh MM; Farrell GC; Lee SP; Savard C Cholesterol crystallization within hepatocyte lipid droplets and its role in murine NASH. J. Lipid Res 2017, 58, 1067–1079. 28404639 10.1194/jlr.M072454 PMC5456359

[99] Duewell P; Kono H; Rayner KJ; Sirois CM; Vladimer G; Bauernfeind FG; Abela GS; Franchi L; Nunez G; Schnurr M; NLRP3 inflammasomes are required for atherogenesis and activated by cholesterol crystals. Nature 2010, 464, 1357–1361. 20428172 10.1038/nature08938 PMC2946640

[100] Rajamaki K; Lappalainen J; Oorni K; Valimaki E; Matikainen S; Kovanen PT; Eklund KK Cholesterol crystals activate the NLRP3 inflammasome in human macrophages: A novel link between cholesterol metabolism and inflammation. PLoS ONE 2010, 5, e11765. 20668705 10.1371/journal.pone.0011765 PMC2909263

[101] Miller YI; Choi SH; Wiesner P; Fang L; Harkewicz R; Hartvigsen K; Boullier A; Gonen A; Diehl CJ; Que X; Oxidation-specific epitopes are danger-associated molecular patterns recognized by pattern recognition receptors of innate immunity. Circ. Res 2011, 108, 235–248. 21252151 10.1161/CIRCRESAHA.110.223875 PMC3075542

[102] Canbay A; Feldstein AE; Higuchi H; Werneburg N; Grambihler A; Bronk SF; Gores GJ Kupffer cell engulfment of apoptotic bodies stimulates death ligand and cytokine expression. Hepatology 2003, 38, 1188–1198. 14578857 10.1053/jhep.2003.50472

[103] Hirsova P; Ibrahim SH; Krishnan A; Verma VK; Bronk SF; Werneburg NW; Charlton MR; Shah VH; Malhi H; Gores GJ Lipid-Induced Signaling Causes Release of Inflammatory Extracellular Vesicles from Hepatocytes. Gastroenterology 2016, 150, 956–967. 26764184 10.1053/j.gastro.2015.12.037 PMC4808464

[104] Wang J; Wu Z; Xia M; Salas SS; Ospina JA; Buist-Homan M; Harmsen MC; Moshage H Extracellular vesicles derived from liver sinusoidal endothelial cells inhibit the activation of hepatic stellate cells and Kupffer cells in vitro. Biochim. Biophys. Acta Mol. Basis Dis 2024, 1870, 167020. 38244390 10.1016/j.bbadis.2024.167020

[105] Mogensen TH Pathogen recognition and inflammatory signaling in innate immune defenses. Clin. Microbiol. Rev 2009, 22, 240–273. 19366914 10.1128/CMR.00046-08 PMC2668232

[106] Frasinariu OE; Ceccarelli S; Alisi A; Moraru E; Nobili V Gut-liver axis and fibrosis in nonalcoholic fatty liver disease: An input for novel therapies. Dig. Liver Dis 2013, 45, 543–551. 23280158 10.1016/j.dld.2012.11.010

[107] Budick-Harmelin N; Dudas J; Demuth J; Madar Z; Ramadori G; Tirosh O Triglycerides potentiate the inflammatory response in rat Kupffer cells. Antioxid. Redox Signal 2008, 10, 2009–2022. 18710323 10.1089/ars.2007.1876

[108] Leroux A; Ferrere G; Godie V; Cailleux F; Renoud ML; Gaudin F; Naveau S; Prevot S; Makhzami S; Perlemuter G; Toxic lipids stored by Kupffer cells correlates with their pro-inflammatory phenotype at an early stage of steatohepatitis. J. Hepatol 2012, 57, 141–149. 22425624 10.1016/j.jhep.2012.02.028

[109] Mills CD; Kincaid K; Alt JM; Heilman MJ; Hill AM M-1/M-2 macrophages and the Th1/Th2 paradigm. J. Immunol 2000, 164, 6166–6173. 28923981 10.4049/jimmunol.1701141

[110] Mosmann TR; Coffman RL TH1 and TH2 cells: Different patterns of lymphokine secretion lead to different functional properties. Annu. Rev. Immunol 1989, 7, 145–173. 2523712 10.1146/annurev.iy.07.040189.001045

[111] Poltorak A; Smirnova I; He X; Liu MY; Van Huffel C; McNally O; Birdwell D; Alejos E; Silva M; Du X; Genetic and physical mapping of the Lps locus: Identification of the toll-4 receptor as a candidate gene in the critical region. Blood Cells Mol. Dis 1998, 24, 340–355. 10087992 10.1006/bcmd.1998.0201

[112] Yang H; Wang H; Ju Z; Ragab AA; Lundback P; Long W; Valdes-Ferrer SI; He M; Pribis JP; Li J; MD-2 is required for disulfide HMGB1-dependent TLR4 signaling. J. Exp. Med 2015, 212, 5–14. 25559892 10.1084/jem.20141318 PMC4291531

[113] Jiang D; Liang J; Fan J; Yu S; Chen S; Luo Y; Prestwich GD; Mascarenhas MM; Garg HG; Quinn DA; Regulation of lung injury and repair by Toll-like receptors and hyaluronan. Nat. Med 2005, 11, 1173–1179. 16244651 10.1038/nm1315

[114] Chen XX; Tang L; Fu YM; Wang Y; Han ZH; Meng JG Paralemmin-3 contributes to lipopolysaccharide-induced inflammatory response and is involved in lipopolysaccharide-Toll-like receptor-4 signaling in alveolar macrophages. Int. J. Mol. Med 2017, 40, 1921–1931. 29039447 10.3892/ijmm.2017.3161

[115] Suganami T; Mieda T; Itoh M; Shimoda Y; Kamei Y; Ogawa Y Attenuation of obesity-induced adipose tissue inflammation in C3H/HeJ mice carrying a Toll-like receptor 4 mutation. Biochem. Biophys. Res. Commun 2007, 354, 45–49. 17210129 10.1016/j.bbrc.2006.12.190

[116] Csak T; Velayudham A; Hritz I; Petrasek J; Levin I; Lippai D; Catalano D; Mandrekar P; Dolganiuc A; Kurt-Jones E; Deficiency in myeloid differentiation factor-2 and toll-like receptor 4 expression attenuates nonalcoholic steatohepatitis and fibrosis in mice. Am. J. Physiol. Gastrointest. Liver Physiol 2011, 300, G433–G441. 21233280 10.1152/ajpgi.00163.2009 PMC3302188

[117] Yang B; Luo W; Wang M; Tang Y; Zhu W; Jin L; Wang M; Wang Y; Zhang Y; Zuo W; Macrophage-specific MyD88 deletion and pharmacological inhibition prevents liver damage in non-alcoholic fatty liver disease via reducing inflammatory response. Biochim. Biophys. Acta Mol. Basis Dis 2022, 1868, 166480. 35811033 10.1016/j.bbadis.2022.166480

[118] Gong J; Li J; Dong H; Chen G; Qin X; Hu M; Yuan F; Fang K; Wang D; Jiang S; Inhibitory effects of berberine on proinflammatory M1 macrophage polarization through interfering with the interaction between TLR4 and MyD88. BMC Complement. Altern. Med 2019, 19, 314. 31744490 10.1186/s12906-019-2710-6 PMC6862859

[119] Xiang P; Chen T; Mou Y; Wu H; Xie P; Lu G; Gong X; Hu Q; Zhang Y; Ji H NZ suppresses TLR4/NF-kappaB signalings and NLRP3 inflammasome activation in LPS-induced RAW264.7 macrophages. Inflamm. Res 2015, 64, 799–808. 26298161 10.1007/s00011-015-0863-4

[120] Lu H; Wu L; Liu L; Ruan Q; Zhang X; Hong W; Wu S; Jin G; Bai Y Quercetin ameliorates kidney injury and fibrosis by modulating M1/M2 macrophage polarization. Biochem. Pharmacol 2018, 154, 203–212. 29753749 10.1016/j.bcp.2018.05.007

[121] Koperska A; Wesolek A; Moszak M; Szulinska M Berberine in Non-Alcoholic Fatty Liver Disease-A Review. Nutrients 2022, 14, 3459. 36079717 10.3390/nu14173459 PMC9459907

[122] Nie Q; Li M; Huang C; Yuan Y; Liang Q; Ma X; Qiu T; Li J The clinical efficacy and safety of berberine in the treatment of non-alcoholic fatty liver disease: A meta-analysis and systematic review. J. Transl. Med 2024, 22, 225. 38429794 10.1186/s12967-024-05011-2 PMC10908013

[123] Ren S; Ma X; Wang R; Liu H; Wei Y; Wei S; Jing M; Zhao Y Preclinical Evidence of Berberine on Non-Alcoholic Fatty Liver Disease: A Systematic Review and Meta-Analysis of Animal Studies. Front. Pharmacol 2021, 12, 742465. 34566663 10.3389/fphar.2021.742465 PMC8458904

[124] Sotiropoulou M; Katsaros I; Vailas M; Lidoriki I; Papatheodoridis GV; Kostomitsopoulos NG; Valsami G; Tsaroucha A; Schizas D Nonalcoholic fatty liver disease: The role of quercetin and its therapeutic implications. Saudi J. Gastroenterol 2021, 27, 319–330. 34810376 10.4103/sjg.sjg_249_21 PMC8656328

[125] Yang H; Yang T; Heng C; Zhou Y; Jiang Z; Qian X; Du L; Mao S; Yin X; Lu Q Quercetin improves nonalcoholic fatty liver by ameliorating inflammation, oxidative stress, and lipid metabolism in db/db mice. Phytother. Res 2019, 33, 3140–3152. 31452288 10.1002/ptr.6486

[126] Qi QR; Yang ZM Regulation and function of signal transducer and activator of transcription 3. World J. Biol. Chem 2014, 5, 231–239. 24921012 10.4331/wjbc.v5.i2.231 PMC4050116

[127] Wang N; Liang H; Zen K Molecular mechanisms that influence the macrophage m1–m2 polarization balance. Front. Immunol 2014, 5, 614. 25506346 10.3389/fimmu.2014.00614 PMC4246889

[128] Sica A; Mantovani A Macrophage plasticity and polarization: In vivo veritas. J. Clin. Investig 2012, 122, 787–795. 22378047 10.1172/JCI59643 PMC3287223

[129] He Y; Gao Y; Zhang Q; Zhou G; Cao F; Yao S IL-4 Switches Microglia/macrophage M1/M2 Polarization and Alleviates Neurological Damage by Modulating the JAK1/STAT6 Pathway Following ICH. Neuroscience 2020, 437, 161–171. 32224230 10.1016/j.neuroscience.2020.03.008

[130] Quero L; Tiaden AN; Hanser E; Roux J; Laski A; Hall J; Kyburz D miR-221–3p Drives the Shift of M2-Macrophages to a Pro-Inflammatory Function by Suppressing JAK3/STAT3 Activation. Front. Immunol 2019, 10, 3087. 32047494 10.3389/fimmu.2019.03087 PMC6996464

[131] Travis MA; Sheppard D TGF-beta activation and function in immunity. Annu. Rev. Immunol 2014, 32, 51–82. 24313777 10.1146/annurev-immunol-032713-120257 PMC4010192

[132] Wang L; Li Y; Wang X; Wang P; Essandoh K; Cui S; Huang W; Mu X; Liu Z; Wang Y; GDF3 Protects Mice against Sepsis-Induced Cardiac Dysfunction and Mortality by Suppression of Macrophage Pro-Inflammatory Phenotype. Cells 2020, 9, 120. 31947892 10.3390/cells9010120 PMC7017037

[133] Fang Y; Jin W; Guo Z; Hao J Quercetin Alleviates Asthma-Induced Airway Inflammation and Remodeling through Downregulating Periostin via Blocking TGF-beta1/Smad Pathway. Pharmacology 2023, 108, 432–443. 37343534 10.1159/000530703

[134] Wahli W; Michalik L PPARs at the crossroads of lipid signaling and inflammation. Trends Endocrinol. Metab 2012, 23, 351–363. 22704720 10.1016/j.tem.2012.05.001

[135] Delerive P; De Bosscher K; Vanden Berghe W; Fruchart JC; Haegeman G; Staels B DNA binding-independent induction of IkappaBalpha gene transcription by PPARalpha. Mol. Endocrinol 2002, 16, 1029–1039. 11981037 10.1210/mend.16.5.0826

[136] Hou Y; Moreau F; Chadee K PPARgamma is an E3 ligase that induces the degradation of NFkappaB/p65. Nat. Commun 2012, 3, 1300. 23250430 10.1038/ncomms2270

[137] Toobian D; Ghosh P; Katkar GD Parsing the Role of PPARs in Macrophage Processes. Front. Immunol 2021, 12, 783780. 35003101 10.3389/fimmu.2021.783780 PMC8727354

[138] Yu L; Gao Y; Aaron N; Qiang L A glimpse of the connection between PPARgamma and macrophage. Front. Pharmacol 2023, 14, 1254317. 37701041 10.3389/fphar.2023.1254317 PMC10493289

[139] Chinetti-Gbaguidi G; Staels B PPARbeta in macrophages and atherosclerosis. Biochimie 2017, 136, 59–64. 28011212 10.1016/j.biochi.2016.12.008

[140] Cheng L; Liu D; Gao S PPARA ameliorates sepsis-induced myocardial injury via promoting macrophage M2 polarization by interacting with DUSP1. Regen Ther. 2024, 26, 33–41. 38798745 10.1016/j.reth.2024.04.017 PMC11126881

[141] Luo W; Xu Q; Wang Q; Wu H; Hua J Effect of modulation of PPAR-gamma activity on Kupffer cells M1/M2 polarization in the development of non-alcoholic fatty liver disease. Sci. Rep 2017, 7, 44612. 28300213 10.1038/srep44612 PMC5353732

[142] Bouhlel MA; Derudas B; Rigamonti E; Dievart R; Brozek J; Haulon S; Zawadzki C; Jude B; Torpier G; Marx N; PPARgamma activation primes human monocytes into alternative M2 macrophages with anti-inflammatory properties. Cell Metab. 2007, 6, 137–143. 17681149 10.1016/j.cmet.2007.06.010

[143] Odegaard JI; Ricardo-Gonzalez RR; Goforth MH; Morel CR; Subramanian V; Mukundan L; Red Eagle A; Vats D; Brombacher F; Ferrante AW; Macrophage-specific PPARgamma controls alternative activation and improves insulin resistance. Nature 2007, 447, 1116–1120. 17515919 10.1038/nature05894 PMC2587297

[144] Wu D; Molofsky AB; Liang HE; Ricardo-Gonzalez RR; Jouihan HA; Bando JK; Chawla A; Locksley RM Eosinophils sustain adipose alternatively activated macrophages associated with glucose homeostasis. Science 2011, 332, 243–247. 21436399 10.1126/science.1201475 PMC3144160

[145] Wang C; Ma C; Gong L; Guo Y; Fu K; Zhang Y; Zhou H; Li Y Macrophage Polarization and Its Role in Liver Disease. Front. Immunol 2021, 12, 803037. 34970275 10.3389/fimmu.2021.803037 PMC8712501

[146] Qian M; Wang S; Guo X; Wang J; Zhang Z; Qiu W; Gao X; Chen Z; Xu J; Zhao R; Hypoxic glioma-derived exosomes deliver microRNA-1246 to induce M2 macrophage polarization by targeting TERF2IP via the STAT3 and NF-kappaB pathways. Oncogene 2020, 39, 428–442. 31485019 10.1038/s41388-019-0996-y

[147] Pan Y; Hui X; Hoo RLC; Ye D; Chan CYC; Feng T; Wang Y; Lam KSL; Xu A Adipocyte-secreted exosomal microRNA-34a inhibits M2 macrophage polarization to promote obesity-induced adipose inflammation. J. Clin. Investig 2019, 129, 834–849. 30667374 10.1172/JCI123069 PMC6355214

[148] Singla RD; Wang J; Singla DK Regulation of Notch 1 signaling in THP-1 cells enhances M2 macrophage differentiation. Am. J. Physiol. Heart Circ. Physiol 2014, 307, H1634–H1642. 25260616 10.1152/ajpheart.00896.2013 PMC4255013

[149] Byles V; Covarrubias AJ; Ben-Sahra I; Lamming DW; Sabatini DM; Manning BD; Horng T The TSC-mTOR pathway regulates macrophage polarization. Nat. Commun 2013, 4, 2834. 24280772 10.1038/ncomms3834 PMC3876736

[150] Yu T; Gao M; Yang P; Liu D; Wang D; Song F; Zhang X; Liu Y Insulin promotes macrophage phenotype transition through PI3K/Akt and PPAR-gamma signaling during diabetic wound healing. J. Cell. Physiol 2019, 234, 4217–4231. 30132863 10.1002/jcp.27185

[151] Zhang Y; Huang T; Jiang L; Gao J; Yu D; Ge Y; Lin S MCP-induced protein 1 attenuates sepsis-induced acute lung injury by modulating macrophage polarization via the JNK/c-Myc pathway. Int. Immunopharmacol 2019, 75, 105741. 31323531 10.1016/j.intimp.2019.105741

[152] Yang Y; Ni M; Zong R; Yu M; Sun Y; Li J; Chen P; Li C Targeting Notch1-YAP Circuit Reprograms Macrophage Polarization and Alleviates Acute Liver Injury in Mice. Cell. Mol. Gastroenterol. Hepatol 2023, 15, 1085–1104. 36706917 10.1016/j.jcmgh.2023.01.002 PMC10036742

[153] Panwar V; Singh A; Bhatt M; Tonk RK; Azizov S; Raza AS; Sengupta S; Kumar D; Garg M Multifaceted role of mTOR (mammalian target of rapamycin) signaling pathway in human health and disease. Signal Transduct. Target. Ther 2023, 8, 375. 37779156 10.1038/s41392-023-01608-z PMC10543444

[154] Pan H; O’Brien TF; Zhang P; Zhong XP The role of tuberous sclerosis complex 1 in regulating innate immunity. J. Immunol 2012, 188, 3658–3666. 22412198 10.4049/jimmunol.1102187 PMC3324625

[155] Zhu L; Yang T; Li L; Sun L; Hou Y; Hu X; Zhang L; Tian H; Zhao Q; Peng J; TSC1 controls macrophage polarization to prevent inflammatory disease. Nat. Commun 2014, 5, 4696. 25175012 10.1038/ncomms5696

[156] Jiang H; Westerterp M; Wang C; Zhu Y; Ai D Macrophage mTORC1 disruption reduces inflammation and insulin resistance in obese mice. Diabetologia 2014, 57, 2393–2404. 25120095 10.1007/s00125-014-3350-5

[157] Ai D; Jiang H; Westerterp M; Murphy AJ; Wang M; Ganda A; Abramowicz S; Welch C; Almazan F; Zhu Y; Disruption of mammalian target of rapamycin complex 1 in macrophages decreases chemokine gene expression and atherosclerosis. Circ. Res 2014, 114, 1576–1584. 24687132 10.1161/CIRCRESAHA.114.302313 PMC4058053

[158] Maina V; Sutti S; Locatelli I; Vidali M; Mombello C; Bozzola C; Albano E Bias in macrophage activation pattern influences non-alcoholic steatohepatitis (NASH) in mice. Clin. Sci 2012, 122, 545–553. 10.1042/CS20110366 22142284

[159] Wan J; Benkdane M; Teixeira-Clerc F; Bonnafous S; Louvet A; Lafdil F; Pecker F; Tran A; Gual P; Mallat A; M2 Kupffer cells promote M1 Kupffer cell apoptosis: A protective mechanism against alcoholic and nonalcoholic fatty liver disease. Hepatology 2014, 59, 130–142. 23832548 10.1002/hep.26607

[160] Papackova Z; Palenickova E; Dankova H; Zdychova J; Skop V; Kazdova L; Cahova M Kupffer cells ameliorate hepatic insulin resistance induced by high-fat diet rich in monounsaturated fatty acids: The evidence for the involvement of alternatively activated macrophages. Nutr. Metab 2012, 9, 22. 10.1186/1743-7075-9-22 PMC3348013 22439764

[161] Odegaard JI; Ricardo-Gonzalez RR; Red Eagle A; Vats D; Morel CR; Goforth MH; Subramanian V; Mukundan L; Ferrante AW; Chawla A Alternative M2 activation of Kupffer cells by PPARdelta ameliorates obesity-induced insulin resistance. Cell Metab. 2008, 7, 496–507. 18522831 10.1016/j.cmet.2008.04.003 PMC2587370

[162] Rolny C; Mazzone M; Tugues S; Laoui D; Johansson I; Coulon C; Squadrito ML; Segura I; Li X; Knevels E; HRG inhibits tumor growth and metastasis by inducing macrophage polarization and vessel normalization through downregulation of PlGF. Cancer Cell 2011, 19, 31–44. 21215706 10.1016/j.ccr.2010.11.009

[163] Bartneck M; Fech V; Ehling J; Govaere O; Warzecha KT; Hittatiya K; Vucur M; Gautheron J; Luedde T; Trautwein C; Histidine-rich glycoprotein promotes macrophage activation and inflammation in chronic liver disease. Hepatology 2016, 63, 1310–1324. 26699087 10.1002/hep.28418

[164] Rensen SS; Slaats Y; Nijhuis J; Jans A; Bieghs V; Driessen A; Malle E; Greve JW; Buurman WA Increased hepatic myeloperoxidase activity in obese subjects with nonalcoholic steatohepatitis. Am. J. Pathol 2009, 175, 1473–1482. 19729473 10.2353/ajpath.2009.080999 PMC2751544

[165] Hart KM; Fabre T; Sciurba JC; Gieseck RL 3rd; Borthwick LA; Vannella KM; Acciani TH; de Queiroz Prado R; Thompson RW; White S; Type 2 immunity is protective in metabolic disease but exacerbates NAFLD collaboratively with TGF-beta. Sci. Transl. Med 2017, 9, eaal3694. 28659437 10.1126/scitranslmed.aal3694

[166] Nakayama T; Hirahara K; Onodera A; Endo Y; Hosokawa H; Shinoda K; Tumes DJ; Okamoto Y Th2 Cells in Health and Disease. Annu. Rev. Immunol 2017, 35, 53–84. 27912316 10.1146/annurev-immunol-051116-052350

[167] Svendsen P; Graversen JH; Etzerodt A; Hager H; Roge R; Gronbaek H; Christensen EI; Moller HJ; Vilstrup H; Moestrup SK Antibody-Directed Glucocorticoid Targeting to CD163 in M2-type Macrophages Attenuates Fructose-Induced Liver Inflammatory Changes. Mol. Ther. Methods Clin. Dev 2017, 4, 50–61. 28344991 10.1016/j.omtm.2016.11.004 PMC5363319

[168] Guillot A; Tacke F Liver Macrophages: Old Dogmas and New Insights. Hepatol. Commun 2019, 3, 730–743. 31168508 10.1002/hep4.1356 PMC6545867

[169] Daemen S; Gainullina A; Kalugotla G; He L; Chan MM; Beals JW; Liss KH; Klein S; Feldstein AE; Finck BN; Dynamic Shifts in the Composition of Resident and Recruited Macrophages Influence Tissue Remodeling in NASH. Cell Rep. 2021, 34, 108626. 33440159 10.1016/j.celrep.2020.108626 PMC7877246

[170] Bonnardel J; T’Jonck W; Gaublomme D; Browaeys R; Scott CL; Martens L; Vanneste B; De Prijck S; Nedospasov SA; Kremer A; Stellate Cells, Hepatocytes, and Endothelial Cells Imprint the Kupffer Cell Identity on Monocytes Colonizing the Liver Macrophage Niche. Immunity 2019, 51, 638–654.e639. 31561945 10.1016/j.immuni.2019.08.017 PMC6876284

[171] Mulder K; Patel AA; Kong WT; Piot C; Halitzki E; Dunsmore G; Khalilnezhad S; Irac SE; Dubuisson A; Chevrier M; Cross-tissue single-cell landscape of human monocytes and macrophages in health and disease. Immunity 2021, 54, 1883–1900.e5. 34331874 10.1016/j.immuni.2021.07.007

[172] Khantakova D; Brioschi S; Molgora M Exploring the Impact of TREM2 in Tumor-Associated Macrophages. Vaccines 2022, 10, 943. 35746551 10.3390/vaccines10060943 PMC9227554

[173] Zhou L; Wang M; Guo H; Hou J; Zhang Y; Li M; Wu X; Chen X; Wang L Integrated Analysis Highlights the Immunosuppressive Role of TREM2(+) Macrophages in Hepatocellular Carcinoma. Front. Immunol 2022, 13, 848367. 35359989 10.3389/fimmu.2022.848367 PMC8963870

[174] Colonna M The biology of TREM receptors. Nat. Rev. Immunol 2023, 23, 580–594. 36750615 10.1038/s41577-023-00837-1 PMC9904274

[175] Labiano I; Agirre-Lizaso A; Olaizola P; Echebarria A; Huici-Izagirre M; Olaizola I; Esparza-Baquer A; Sharif O; Hijona E; Milkiewicz P; TREM-2 plays a protective role in cholestasis by acting as a negative regulator of inflammation. J. Hepatol 2022, 77, 991–1004. 35750136 10.1016/j.jhep.2022.05.044

[176] Hendrikx T; Porsch F; Kiss MG; Rajcic D; Papac-Milicevic N; Hoebinger C; Goederle L; Hladik A; Shaw LE; Horstmann H; Soluble TREM2 levels reflect the recruitment and expansion of TREM2(+) macrophages that localize to fibrotic areas and limit NASH. J. Hepatol 2022, 77, 1373–1385. 35750138 10.1016/j.jhep.2022.06.004

[177] Coelho I; Duarte N; Barros A; Macedo MP; Penha-Goncalves C Trem-2 Promotes Emergence of Restorative Macrophages and Endothelial Cells during Recovery from Hepatic Tissue Damage. Front. Immunol 2020, 11, 616044. 33628208 10.3389/fimmu.2020.616044 PMC7897679

[178] Krenkel O; Hundertmark J; Abdallah AT; Kohlhepp M; Puengel T; Roth T; Branco DPP; Mossanen JC; Luedde T; Trautwein C; Myeloid cells in liver and bone marrow acquire a functionally distinct inflammatory phenotype during obesity-related steatohepatitis. Gut 2020, 69, 551–563. 31076404 10.1136/gutjnl-2019-318382

[179] Halpern KB; Shenhav R; Matcovitch-Natan O; Toth B; Lemze D; Golan M; Massasa EE; Baydatch S; Landen S; Moor AE; Single-cell spatial reconstruction reveals global division of labour in the mammalian liver. Nature 2017, 542, 352–356. 28166538 10.1038/nature21065 PMC5321580

[180] Satija R; Farrell JA; Gennert D; Schier AF; Regev A Spatial reconstruction of single-cell gene expression data. Nat. Biotechnol 2015, 33, 495–502. 25867923 10.1038/nbt.3192 PMC4430369

[181] Guillot A; Tacke F Spatial dimension of macrophage heterogeneity in liver diseases. eGastroenterology 2023, 1, e000003. 10.1136/egastro-2023-000003 PMC11770455 39944246

[182] Guilliams M; Bonnardel J; Haest B; Vanderborght B; Wagner C; Remmerie A; Bujko A; Martens L; Thone T; Browaeys R; Spatial proteogenomics reveals distinct and evolutionarily conserved hepatic macrophage niches. Cell 2022, 185, 379–396.e338. 35021063 10.1016/j.cell.2021.12.018 PMC8809252

[183] Gola A; Dorrington MG; Speranza E; Sala C; Shih RM; Radtke AJ; Wong HS; Baptista AP; Hernandez JM; Castellani G; Commensal-driven immune zonation of the liver promotes host defence. Nature 2021, 589, 131–136. 33239787 10.1038/s41586-020-2977-2 PMC8691525

[184] Andrews TS; Atif J; Liu JC; Perciani CT; Ma XZ; Thoeni C; Slyper M; Eraslan G; Segerstolpe A; Manuel J; Single-Cell, Single-Nucleus, and Spatial RNA Sequencing of the Human Liver Identifies Cholangiocyte and Mesenchymal Heterogeneity. Hepatol. Commun 2022, 6, 821–840. 34792289 10.1002/hep4.1854 PMC8948611

[185] Camp JG; Sekine K; Gerber T; Loeffler-Wirth H; Binder H; Gac M; Kanton S; Kageyama J; Damm G; Seehofer D; Multilineage communication regulates human liver bud development from pluripotency. Nature 2017, 546, 533–538. 28614297 10.1038/nature22796

[186] Cavalli M; Diamanti K; Pan G; Spalinskas R; Kumar C; Deshmukh AS; Mann M; Sahlen P; Komorowski J; Wadelius C A Multi-Omics Approach to Liver Diseases: Integration of Single Nuclei Transcriptomics with Proteomics and HiCap Bulk Data in Human Liver. OMICS 2020, 24, 180–194. 32181701 10.1089/omi.2019.0215 PMC7185313

[187] Dudek M; Pfister D; Donakonda S; Filpe P; Schneider A; Laschinger M; Hartmann D; Huser N; Meiser P; Bayerl F; Auto-aggressive CXCR6(+) CD8 T cells cause liver immune pathology in NASH. Nature 2021, 592, 444–449. 33762736 10.1038/s41586-021-03233-8

[188] Hou X; Yang Y; Li P; Zeng Z; Hu W; Zhe R; Liu X; Tang D; Ou M; Dai Y Integrating Spatial Transcriptomics and Single-Cell RNA-seq Reveals the Gene Expression Profling of the Human Embryonic Liver. Front. Cell Dev. Biol 2021, 9, 652408. 34095116 10.3389/fcell.2021.652408 PMC8173368

[189] Payen VL; Lavergne A; Alevra Sarika N; Colonval M; Karim L; Deckers M; Najimi M; Coppieters W; Charloteaux B; Sokal EM; Single-cell RNA sequencing of human liver reveals hepatic stellate cell heterogeneity. JHEP Rep. 2021, 3, 100278. 34027339 10.1016/j.jhepr.2021.100278 PMC8121977

[190] Wang ZY; Keogh A; Waldt A; Cuttat R; Neri M; Zhu S; Schuierer S; Ruchti A; Crochemore C; Knehr J; Single-cell and bulk transcriptomics of the liver reveals potential targets of NASH with fibrosis. Sci. Rep 2021, 11, 19396. 34588551 10.1038/s41598-021-98806-y PMC8481490

[191] Filliol A; Saito Y; Nair A; Dapito DH; Yu LX; Ravichandra A; Bhattacharjee S; Affo S; Fujiwara N; Su H; Opposing roles of hepatic stellate cell subpopulations in hepatocarcinogenesis. Nature 2022, 610, 356–365. 36198802 10.1038/s41586-022-05289-6 PMC9949942

[192] Kotsiliti E; Leone V; Schuehle S; Govaere O; Li H; Wolf MJ; Horvatic H; Bierwirth S; Hundertmark J; Inverso D; Intestinal B cells license metabolic T-cell activation in NASH microbiota/antigen-independently and contribute to fibrosis by IgA-FcR signalling. J. Hepatol 2023, 79, 296–313. 37224925 10.1016/j.jhep.2023.04.037 PMC10360918

[193] Wang S; Li K; Pickholz E; Dobie R; Matchett KP; Henderson NC; Carrico C; Driver I; Borch Jensen M; Chen L; An autocrine signaling circuit in hepatic stellate cells underlies advanced fibrosis in nonalcoholic steatohepatitis. Sci. Transl. Med 2023, 15, eadd3949. 36599008 10.1126/scitranslmed.add3949 PMC10686705

[194] Xiao Y; Batmanov K; Hu W; Zhu K; Tom AY; Guan D; Jiang C; Cheng L; McCright SJ; Yang EC; Hepatocytes demarcated by EphB2 contribute to the progression of nonalcoholic steatohepatitis. Sci. Transl. Med 2023, 15, eadc9653. 36753562 10.1126/scitranslmed.adc9653 PMC10234568

[195] Guillot A; Winkler M; Silva Afonso M; Aggarwal A; Lopez D; Berger H; Kohlhepp MS; Liu H; Ozdirik B; Eschrich J; Mapping the hepatic immune landscape identifies monocytic macrophages as key drivers of steatohepatitis and cholangiopathy progression. Hepatology 2023, 78, 150–166. 36630995 10.1097/HEP.0000000000000270

[196] Ait Ahmed Y; Lafdil F; Tacke F Ambiguous Pathogenic Roles of Macrophages in Alcohol-Associated Liver Diseases. Hepat. Med 2023, 15, 113–127. 37753346 10.2147/HMER.S326468 PMC10519224

[197] Peiseler M; Araujo David B; Zindel J; Surewaard BGJ; Lee WY; Heymann F; Nusse Y; Castanheira FVS; Shim R; Guillot A; Kupffer cell-like syncytia replenish resident macrophage function in the fibrotic liver. Science 2023, 381, eabq5202. 37676943 10.1126/science.abq5202

[198] Stienstra R; Saudale F; Duval C; Keshtkar S; Groener JE; van Rooijen N; Staels B; Kersten S; Muller M Kupffer cells promote hepatic steatosis via interleukin-1beta-dependent suppression of peroxisome proliferator-activated receptor alpha activity. Hepatology 2010, 51, 511–522. 20054868 10.1002/hep.23337

[199] Huang W; Metlakunta A; Dedousis N; Zhang P; Sipula I; Dube JJ; Scott DK; O’Doherty RM Depletion of liver Kupffer cells prevents the development of diet-induced hepatic steatosis and insulin resistance. Diabetes 2010, 59, 347–357. 19934001 10.2337/db09-0016 PMC2809951

[200] Kudo H; Takahara T; Yata Y; Kawai K; Zhang W; Sugiyama T Lipopolysaccharide triggered TNF-alpha-induced hepatocyte apoptosis in a murine non-alcoholic steatohepatitis model. J. Hepatol 2009, 51, 168–175. 19446916 10.1016/j.jhep.2009.02.032

[201] Ratziu V; Sanyal A; Harrison SA; Wong VW; Francque S; Goodman Z; Aithal GP; Kowdley KV; Seyedkazemi S; Fischer L; Cenicriviroc Treatment for Adults with Nonalcoholic Steatohepatitis and Fibrosis: Final Analysis of the Phase 2b CENTAUR Study. Hepatology 2020, 72, 892–905. 31943293 10.1002/hep.31108

[202] Harrison SA; Marri SR; Chalasani N; Kohli R; Aronstein W; Thompson GA; Irish W; Miles MV; Xanthakos SA; Lawitz E; Randomised clinical study: GR-MD-02, a galectin-3 inhibitor, vs. placebo in patients having non-alcoholic steatohepatitis with advanced fibrosis. Aliment. Pharmacol. Ther 2016, 44, 1183–1198. 27778367 10.1111/apt.13816

[203] Friedman SL; Ratziu V; Harrison SA; Abdelmalek MF; Aithal GP; Caballeria J; Francque S; Farrell G; Kowdley KV; Craxi A; A randomized, placebo-controlled trial of cenicriviroc for treatment of nonalcoholic steatohepatitis with fibrosis. Hepatology 2018, 67, 1754–1767. 28833331 10.1002/hep.29477 PMC5947654

[204] Hsu DK; Yang RY; Pan Z; Yu L; Salomon DR; Fung-Leung WP; Liu FT Targeted disruption of the galectin-3 gene results in attenuated peritoneal inflammatory responses. Am. J. Pathol 2000, 156, 1073–1083. 10702423 10.1016/S0002-9440(10)64975-9 PMC1876862

[205] Liu FT; Hsu DK; Zuberi RI; Kuwabara I; Chi EY; Henderson WR Jr. Expression and function of galectin-3, a beta-galactoside-binding lectin, in human monocytes and macrophages. Am. J. Pathol 1995, 147, 1016–1028. 7573347 PMC1871012

[206] Hughes RC Galectins as modulators of cell adhesion. Biochimie 2001, 83, 667–676. 11522396 10.1016/s0300-9084(01)01289-5

[207] Di Lella S; Sundblad V; Cerliani JP; Guardia CM; Estrin DA; Vasta GR; Rabinovich GA When galectins recognize glycans: From biochemistry to physiology and back again. Biochemistry 2011, 50, 7842–7857. 21848324 10.1021/bi201121m PMC3429939

[208] Iacobini C; Menini S; Ricci C; Blasetti Fantauzzi C; Scipioni A; Salvi L; Cordone S; Delucchi F; Serino M; Federici M; Galectin-3 ablation protects mice from diet-induced NASH: A major scavenging role for galectin-3 in liver. J. Hepatol 2011, 54, 975–983. 21145823 10.1016/j.jhep.2010.09.020

[209] Jeftic I; Jovicic N; Pantic J; Arsenijevic N; Lukic ML; Pejnovic N Galectin-3 Ablation Enhances Liver Steatosis, but Attenuates Inflammation and IL-33-Dependent Fibrosis in Obesogenic Mouse Model of Nonalcoholic Steatohepatitis. Mol. Med 2015, 21, 453–465. 26018806 10.2119/molmed.2014.00178 PMC4559528

[210] Traber PG; Zomer E Therapy of experimental NASH and fibrosis with galectin inhibitors. PLoS ONE 2013, 8, e83481. 24367597 10.1371/journal.pone.0083481 PMC3867460

[211] Chalasani N; Abdelmalek MF; Garcia-Tsao G; Vuppalanchi R; Alkhouri N; Rinella M; Noureddin M; Pyko M; Shiffman M; Sanyal A; Effects of Belapectin, an Inhibitor of Galectin-3, in Patients with Nonalcoholic Steatohepatitis with Cirrhosis and Portal Hypertension. Gastroenterology 2020, 158, 1334–1345.e1335. 31812510 10.1053/j.gastro.2019.11.296

[212] Noureddin M MASH clinical trials and drugs pipeline: An impending tsunami. Hepatology 2024. 10.1097/HEP.0000000000000860 38502810

[213] Jiang C; Li P; Ruan X; Ma Y; Kawai K; Suemizu H; Cao H Comparative Transcriptomics Analyses in Livers of Mice, Humans, and Humanized Mice Define Human-Specific Gene Networks. Cells 2020, 9, 2566. 33266321 10.3390/cells9122566 PMC7761003

[214] Teufel A; Itzel T; Erhart W; Brosch M; Wang XY; Kim YO; von Schonfels W; Herrmann A; Bruckner S; Stickel F; Comparison of Gene Expression Patterns between Mouse Models of Nonalcoholic Fatty Liver Disease and Liver Tissues from Patients. Gastroenterology 2016, 151, 513–525.e510. 27318147 10.1053/j.gastro.2016.05.051

[215] Saldarriaga OA; Wanninger TG; Arroyave E; Gosnell J; Krishnan S; Oneka M; Bao D; Millian DE; Kueht ML; Moghe A; Heterogeneity in intrahepatic macrophage populations and druggable target expression in patients with steatotic liver disease-related fibrosis. JHEP Rep. 2024, 6, 100958. 38162144 10.1016/j.jhepr.2023.100958 PMC10757256

[216] Guillot A; Tacke F Liver macrophages revisited: The expanding universe of versatile responses in a spatiotemporal context. Hepatol. Commun 2024, 8, e0491. 38967563 10.1097/HC9.0000000000000491 PMC11227356

